# Pharmacokinetics of Pullulan–Dexamethasone Conjugates in Retinal Drug Delivery

**DOI:** 10.3390/pharmaceutics14010012

**Published:** 2021-12-21

**Authors:** Eva Kicková, Amir Sadeghi, Jooseppi Puranen, Shirin Tavakoli, Merve Sen, Veli-Pekka Ranta, Blanca Arango-Gonzalez, Sylvia Bolz, Marius Ueffing, Stefano Salmaso, Paolo Caliceti, Elisa Toropainen, Marika Ruponen, Arto Urtti

**Affiliations:** 1 Department of Pharmaceutical and Pharmacological Sciences, University of Padova, Via F. Marzolo 5, 35131 Padova, Italy; eva.kickova@yahoo.com (E.K.); stefano.salmaso@unipd.it (S.S.); paolo.caliceti@unipd.it (P.C.); 2 School of Pharmacy, Faculty of Health Sciences, University of Eastern Finland, Yliopistonranta 1C, 70211 Kuopio, Finland; amir.sadeghi@uef.fi (A.S.); jooseppi.puranen@uef.fi (J.P.); veli-pekka.ranta@uef.fi (V.-P.R.); elisa.toropainen@uef.fi (E.T.); marika.ruponen@uef.fi (M.R.); 3 Drug Research Program, Faculty of Pharmacy, University of Helsinki, Viikinkaari 5E, 00710 Helsinki, Finland; shirin.tavakoli@helsinki.fi; 4 Centre for Ophthalmology, Institute for Ophthalmic Research, University of Tübingen, Elfriede-Aulhorn-Str. 7, D-72076 Tübingen, Germany; merve.sen@uni-tuebingen.de (M.S.); blanca.arango-gonzalez@klinikum.uni-tuebingen.de (B.A.-G.); sylvia.bolz@uni-tuebingen.de (S.B.); mue@klinikum.uni-tuebingen.de (M.U.); 5 Institute of Chemistry, St. Petersburg State University, Petergof, Universitetskii pr. 26, 198504 St. Petersburg, Russia

**Keywords:** pullulan, dexamethasone, conjugate, retinal drug delivery, ocular fluorophotometry, optical coherence tomography, pharmacokinetics

## Abstract

The treatment of retinal diseases by intravitreal injections requires frequent administration unless drug delivery systems with long retention and controlled release are used. In this work, we focused on pullulan (≈67 kDa) conjugates of dexamethasone as therapeutic systems for intravitreal administration. The pullulan–dexamethasone conjugates self-assemble into negatively charged nanoparticles (average size 326 ± 29 nm). Intravitreal injections of pullulan and pullulan–dexamethasone were safe in mouse, rat and rabbit eyes. Fluorescently labeled pullulan particles showed prolonged retention in the vitreous and they were almost completely eliminated via aqueous humor outflow. Pullulan conjugates also distributed to the retina via Müller glial cells when tested in ex vivo retina explants and in vivo. Pharmacokinetic simulations showed that pullulan–dexamethasone conjugates may release free and active dexamethasone in the vitreous humor for over 16 days, even though a large fraction of dexamethasone may be eliminated from the eye as bound pullulan–dexamethasone. We conclude that pullulan based drug conjugates are promising intravitreal drug delivery systems as they may reduce injection frequency and deliver drugs into the retinal cells.

## 1. Introduction

Intravitreal injection is the most important mode of drug administration in the treatment of retinal diseases. In particular, anti-inflammatory drugs (e.g., corticosteroids) [1,2,3] and anti-neovascular inhibitors of vascular endothelial growth factor (VEGF) (antibodies, Fab-fragments, soluble receptors and aptamers) are widely used in clinics [4,5,6,7,8]. In general, intravitreal injections are safe, but frequent injections may result in reduced patient compliance and some rare, but serious, adverse effects (e.g., infection and retinal detachment) [5,9]. Implants with prolonged ocular residence and controlled release have been developed to prolong injection intervals of dexamethasone (e.g., Ozurdex) [1,3,9]. On the other hand, delivery systems for trafficking therapeutics to the retinal cells (e.g., intracellularly acting peptides, proteins and nucleic acids) are needed. Therefore, nanoparticles and peptide conjugates have been recently investigated for retinal delivery of dexamethasone and nucleic acids [10,11,12].

Modified polysaccharides are promising candidates for the development of ocular drug delivery systems [13]. For example, pullulan [14], dextran [15], hyaluronic acid [16] and chitosan [17] have been investigated in this respect. Many polysaccharides can be chemically functionalized for optimized drug delivery [13,18] and they can be formulated as macroscopic implants [19,20], gels [21] and nanosized formulations [22]. In most studies, drugs have been physically encapsulated into the polysaccharide formulations, such as polymeric micelles or nanoparticles [13,23,24,25], whereas chemical covalent conjugation technologies have not been applied for ocular in vivo drug delivery with polysaccharides. Recently, the chemical conjugation through hydrazone bond was used to generate peptide-dexamethasone and pullulan–dexamethasone conjugates that were investigated in vitro [10,26]. In contrast to the ocular field, polymeric drug conjugates have been widely investigated to provide site-specificity and extended drug release in some other medical indications [27,28,29,30,31,32].

We have been investigating drug carriers based on pullulan, a fungal extracellular polysaccharide produced by *Aureobasidium pullulans* [33,34]. Inexpensive and conveniently modified pullulan is considered to be a biocompatible polymer. Previously, pullulan has been used as a backbone in the synthesis of bioconjugates for drug delivery to the liver and pancreas [14,27,33,35,36,37]. Conjugation of hydrophobic drugs to pullulan results in self-assembled colloids with drug molecules oriented to the core of the nanoparticles [27,28,33,35,36].

In this study, we investigated dexamethasone conjugates of pullulan that were obtained with recently published synthetic procedures [26]. Dexamethasone was conjugated to pullulan through a hydrazone bond that is expected to control drug release under the acidic intracellular compartments (endosomes or lysosomes). We investigated ocular safety, retinal distribution and ocular pharmacokinetics of fluorescently labelled pullulan–dexamethasone after intravitreal injections into mouse, rat and rabbit eyes. Retinal distribution of the conjugates was also investigated using ex vivo retinas of mice and cows.

## 2. Materials and Methods

Pullulan (67 kDa) was purchased from Hayashibara Biochemical Laboratories, Okayama, Japan. Dexamethasone (DEX) was purchased from Sigma/Merck KgaA, Darmstadt, Germany. Cyanine3 (Cy3) and bodipy (BDP) were purchased from Lumiprobe GmbH, Hannover, Germany.

### 2.1. Synthesis of Pullulan Conjugates

**Figure 1:**
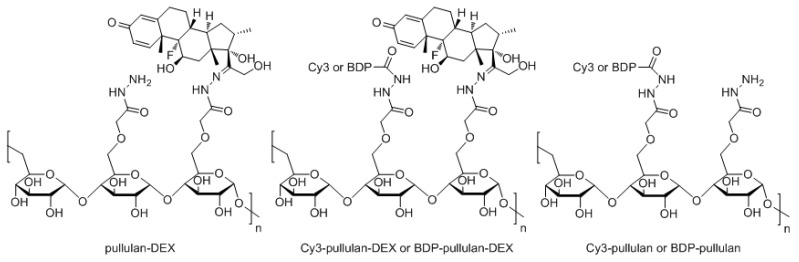
Chemical structures of fluorescently labelled pullulan conjugates. Synthetic details can be found in a recent publication [26] and in Supporting Information SI-1.

Cy3-pullulan-DEX and Cy3-pullulan have recently been prepared and characterized by Kicková et al. [26]. Similar synthetic strategy was applied to prepare versions with green fluorescent label starting from pullulan-DEX, namely BDP-pullulan-DEX and BDP-pullulan (Supporting Information, SI-1). The chemical identity of the conjugates (Figure 1) was confirmed by the NMR spectroscopy (SI-1 and in Kicková et al. [26]).

Stock samples of pullulan-DEX, Cy3-pullulan-DEX, BDP-pullulan-DEX, Cy3-pullulan and BDP-pullulan were generated by dispersing them in mQ water or phosphate buffered saline.

### 2.2. Size and Zeta Potential

Aqueous dispersions of pullulan-DEX, Cy3-pullulan-DEX and BDP-pullulan-DEX were analyzed by dynamic light scattering (DLS) using the Zetasizer Nano ZS (Malvern Instrument Ltd., Malvern, Worcestershire, UK). Zeta potentials were analyzed from 1 mg/mL polymer dispersions in 1 mM phosphate buffer (pH 7.4) at room temperature. All analyses were performed in triplicate.

### 2.3. Endotoxin Tests

The endotoxins in the pullulan conjugate dispersions were determined using Limulus Amebocyte Lysate gel-clot endotoxin assessment kit (Bioscience Lonza, Basel, Switzerland) according to the manufacturer’s instructions. The stock solutions (5 mg/mL) of BDP-pullulan-DEX and BDP-pullulan were prepared in sterile PBS at pH 7.4. These dispersions were further diluted 1:1, 1:2 and 1:4 (*v*/*v*) with sterile endotoxin-free water as duplicates. The conjugate samples, endotoxin standards (1.0, 0.5, 0.25, 0.125, 0.06 and 0.03 EU/mL) and endotoxin-free water were transferred to reaction tubes (100 μL/tube) and reconstituted with Limulus Amebocyte Lysate reagent (100 μL) in each tube. After one-hour incubation at 37 °C each tube was inverted 180 degrees. Formation of firm gel was considered as an indication of endotoxin positivity.

### 2.4. Ex Vivo Retinal Studies

#### 2.4.1. Ex Vivo Mouse Retinal Organ Culture

Six day old (PN6) wild-type mice (C57BL/6) were used in this study. The mice were housed and bred under standard white cyclic lighting with free access to food and water. All mouse procedures were approved by the Tübingen University committee on animal protection (Mitteilung nach §4 Abs. 3 TierSchG Nr. AK 03/20 M) and performed in compliance with the Association for Research in Vision and Ophthalmology ARVO Statement.

The preparation of organotypic retinal culture and maintenance of the retinal explants were performed according to published protocols [38,39,40,41] (for more details see SI-2). The tissues were randomly assigned to the following treatment groups: Cy3-pullulan (1.7 mg/mL), Cy3-pullulan-DEX (0.7, 1.4, and 1.9 mg/mL) and untreated control. The treatments were carefully applied on the top of the retinal tissues (on the ganglion cell layer) using volumes of 15 μL. Six retinal tissues were used for each group. The complete medium (1 mL) under the retina was changed every 48 h and maintained in a humidified atmosphere of 5% CO_2_ at 37 °C for six days.

The dying cells in the retinal explants were monitored using TUNEL assay with in situ cell death detection kit based on conjugated fluorescein isothiocyanate [42]. The percentage of positive cells was derived by dividing the number of positive cells by the total number of outer nuclear layer (ONL) or inner nuclear layer (INL) cells. One-way ANOVA method and Tukey’s multiple comparisons test were selected for the statistical analyses, **** *p* < 0.0001.

#### 2.4.2. Ex Vivo Bovine Vitreo-Retinal Organ Culture

Fresh bovine eyes were obtained from a local slaughterhouse (HKScan Finland Oy, Outokumpu, Turku, Finland). The eyes were transported in carbon dioxide-independent medium at 4 °C (GIBCO, Thermo Fisher Scientific, Dreieich, Germany), cleaned from the connective tissues and dipped shortly into 20% (*v*/*v*) ethanol/water solution. The eyes were kept in carbon dioxide-independent medium at room temperature followed by 10 min incubation at 37 °C prior to dissection.

Vitreo-retinal explants were prepared as reported by Tavakoli et al. [43,44]. The vitreo-retinal explant with intact inner limiting membrane (1–2 cm^2^) was placed onto a Transwell^®^ membrane (75 mm, 0.4 μm pore, Corning Incorporated, Kennebunk, ME, USA) and the supplemented Neurobasal^®^-A medium (GIBCO, Thermo Fisher Scientific, Dreieich, Germany) was added under the membrane. BDP-pullulan-DEX (5 mg/mL) was carefully injected (100 μL) in the vitreous of the vitreo-retinal explant. Injections were performed horizontally to prevent retinal damage and avoid crossing the inner limiting membrane. The vitreo-retinal explant was maintained in a humidified atmosphere containing 5% CO_2_ at 37 °C.

After incubation for 24 h, 20 cryosections of the vitreo-retinal explant were generated. Immunohistochemistry and imaging were performed according to the previously published methods [43,44]. Rabbit anti-collagen IV antibody (Abcam plc., Cambridge, UK) was used for labelling the inner limiting membrane. Hoechst (Thermo Fisher Scientific Inc./Invitrogen™, Carlsbad, CA, USA) stain was used to label the nuclei and Alexa Fluor 568-labelled goat anti-rabbit secondary antibody (Thermo Fisher Scientific Inc./Invitrogen™, Carlsbad, CA, USA) was used to label inner limiting membrane. The images were obtained by confocal microscope (Leica TCS SP8, Leica Microsystems GmbH, Wetzlar, Germany) using 20× (HC PL APO) and 93× (HC PL APO) objectives.

### 2.5. In Vivo Animal Studies

Four months old male pigmented rats (HsdOla/LH), twelve-months old female albino New Zealand White rabbits and two-months old male pigmented mice (C57BL/6J) were used in these studies. The animals were housed under standard white cyclic lighting with free access to food and water. All experiments were designed and conducted in accordance with the guidelines of the ARVO Statement for the Use of Animals in Ophthalmic and Vision Research. All procedures were approved by the Finnish National Animal Experiment Broad (ELLA, Regional State Administrative Agency for Southern Finland), performed under project license (ESAVI-2020-027769) and in compliance with 3Rs principle (replacement, reduction and refinement) monitored by animal-welfare body of University of Eastern Finland Lab Animal Center (UEF LAC).

#### 2.5.1. Safety Studies in Mice

Mice were anesthetized with intraperitoneal injection of 60 mg/kg ketamine (Ketaminol^®^, 50 mg/mL; Pfizer Oy Animal Health, Espoo, Finland) and 0.4 mg/kg medetomidine (Domitor^®^, 1 mg/mL; Orion Pharma, Espoo, Finland). The mouse pupils were dilated with topically applied 0.5% tropicamide (Oftan^®^ Tropicamid, 5 mg/mL; Santen Pharmaceutical Co., Ltd., Tampere, Finland). Under full anaesthesia, volumes of 1 µL of Cy3-pullulan (5 mg/mL) or Cy3-pullulan-DEX (5 mg/mL) in PBS (pH 7.4) were injected intravitreally into mice using Hamilton microinjector (Hamilton Co., Reno, NV, USA). A topical eye drop (Viscotears^®^, Alcon, Finland) was applied after intravitreal injections to prevent dryness of the cornea. Quality of intravitreal injections was confirmed by optical coherence tomography (OCT) and fundus camera (Phoenix MICRON^TM^, Berkeley, CA, USA).

After 24 h the mice were sacrificed, the eyes were removed and incubated in a 4% PFA solution for 2 h. The eyes were stored in 1% PFA solution until further processing of organotypic retinal cultures. The following procedures were performed according to published protocols [38,39,40,41,42] and method described in SI-2.

#### 2.5.2. Ocular Retention and Safety Studies in Rats

Anesthesia was induced in a box using an inhalation system run at 450–500 mL/min air flow and ≈4% of isoflurane purchased from Chanelle Pharma (London, UK). The anesthesia was maintained by 200–250 mL/min air flow containing ≈2% isoflurane. The eye muscles were relaxed with topical instillation of medetomidine. The pupil was dilated by topical instillation of tropicamide and phenylephrine (Oftan^®^ Metaoksedrin, 100 mg/mL; Santen Pharmaceutical Co., Ltd., Tampere, Finland) few minutes before each measurement. The baseline autofluorescence in fluorophotometry (Ocumetrics, Inc., Mountain View, CA, USA) and fundus/OCT images of each eye were captured before intravitreal injections. Local ocular surface anesthesia was induced shortly before intravitreal injections by topical instillation of oxybuprocaine (Oftan^®^ Obucain, 4 mg/mL; Santen Pharmaceutical Co., Ltd., Tampere, Finland).

The injected solutions were prepared in isotonic PBS buffer in a sterile condition. Intravitreal injections of BDP-pullulan (3 µL, 5 mg/mL) and BDP-pullulan-DEX (3 µL, 10 mg/mL) in PBS (pH 7.4) were performed with a Hamilton syringe (Hamilton Co., Reno, NV, USA) equipped with a 34 G needle. BDP-pullulan with 2.2% GPU (glucose per unit, repetition unit in the pullulan chain) modification by BDP (corresponding to 6% *w*/*w*) and BDP-pullulan-DEX with 1.1% GPU modification by BDP (corresponding to 3% *w*/*w*) and 5.2% GPU modification by DEX (corresponding to 10% *w*/*w*) were used in these experiments. Immediately after the intravitreal injections the eyes were topically covered with carbomer hydrogel (Viscotears^®^, 2 mg/g; Dr. Winzer Pharma, Berlin, Germany) to prevent corneal drying. Fundus and OCT images were obtained to check the quality of injections. The procedures of anesthesia, topical drop, muscle relaxant and pupillary dilatant applications were used in all measurements.

#### 2.5.3. Fluorophotometric Studies with Rabbits

The rabbits were anesthetized by s.c. injection of 0.5 mg/kg medetomidine and ketamine (25 mg/kg; Ketaminol^®^, 50 mg/mL; Pfizer Oy Animal Health, Espoo, Finland). The pupils were dilated by using topical tropicamide eye drop. The baseline autofluorescence for each eye was measured before intravitreal injection. Oxybuprocaine was instilled topically as local anesthetic a few minutes before the intravitreal injections.

The intravitreal injection (50 μL) of BDP-pullulan-DEX (10 mg/mL) solution in PBS (pH 7.4) was performed with 31 G needle inserted about 4 mm from the limbus trans-sclerally into the vitreous. Immediately after intravitreal injections, the eyes were topically covered with carbomer hydrogel to prevent corneal dryness.

The experimental measurements were performed under light sedation at various time points post-injection. Medetomidine (0.4 mg/kg) was used as a sedative by s.c. injection and topical tropicamide was used to dilate pupils a few minutes before each measurement. Atipamezole (0.2 mL/kg; Antisedan^®^, 5 mg/mL; Orion Pharma, Espoo, Finland) was used as an antagonist to reverse the sedation by s.c. injection.

The post-injection fluorescence signals were measured from the vitreous and aqueous humor. Autofluorescence was subtracted and the resulting values were converted to BDP-pullulan and BDP-pullulan-DEX concentrations with calibration standards (see SI-3, Figures S2 and S3). The concentrations were used to determine pharmacokinetic parameters (clearance, volume of distribution and half-life) with PKSolver software [45]. One compartment model with first-order elimination rate was used for curve fitting. The nonlinear weighting [1/(observed concentration)^2^] method was used to improve the quality of fitting for terminal time points. More details on in vivo fluorophotometry and pharmacokinetic analyses can be found in our earlier publications [10,15].

#### 2.5.4. Pharmacokinetic Simulations

**Figure 2:**
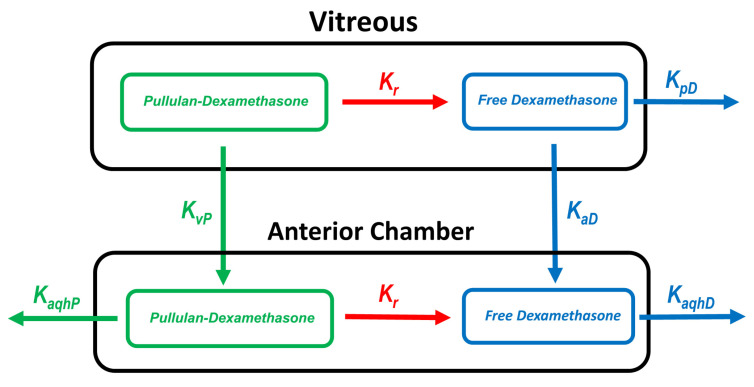
Scheme of the kinetic simulation model. The following parameters were used: K_r_ (first-order release rate constant); K_pD_ (elimination rate constant of free dexamethasone posteriorly from the vitreous); K_aD_ (distribution rate constant of free dexamethasone from the vitreous to the anterior chamber); K_vP_ (distribution rate constant of pullulan–dexamethasone from the vitreous to the anterior chamber); K_aqhP_ (elimination rate constant of pullulan–dexamethasone from the anterior chamber) and K_aqhD_ (elimination constant of dexamethasone from the anterior chamber). For detailed parameter values, see Supporting Information SI-4.

Pharmacokinetic simulations were performed to estimate the elimination routes of polymer conjugate. The experimental values for the vitreal clearance and volume of distribution were used to build the model. The simulated in vivo release rate of dexamethasone was assumed to be similar with the release rate in vitro [26] (SI-4, Figure S4). The simulations for the concentrations of free dexamethasone in the vitreous and aqueous humor were performed for the rat, rabbit and human eyes. The schematic representation of the model is shown in Figure 2. The details are shown in SI-4, Table S1 and Figure S5. For numerical simulations, STELLA^®^ software version. 8.1.1 (isee systems, Lebanon, NH, USA) was used with fourth order Runge–Kutta algorithm.

## 3. Results

### 3.1. Synthesis and Characterization of Pullulan Conjugates

Pullulan is a water-soluble polysaccharide but conjugation of pullulan with dexamethasone (DEX) as a hydrophobic molecule results in an amphiphilic derivative that undergoes self-assembly to nanoparticles. The self-assembled particles of Cy3-pullulan-DEX and BDP-pullulan-DEX were smaller than pullulan-DEX particles and all nanoparticles had negative zeta potentials (Table 1).

The endotoxin level of formulations was measured. All tested concentrations (2.5, 1.25 and 0.63 mg/mL) of fluorescently labelled BDP-pullulan-DEX showed endotoxin levels below 0.03 mEU/μL. Thus, endotoxin levels in the formulations used for in vivo administration are at acceptable levels below 0.2 EU per injection [46,47].

### 3.2. Intravitreal Kinetics of Pullulan Conjugates

**Figure 3:**
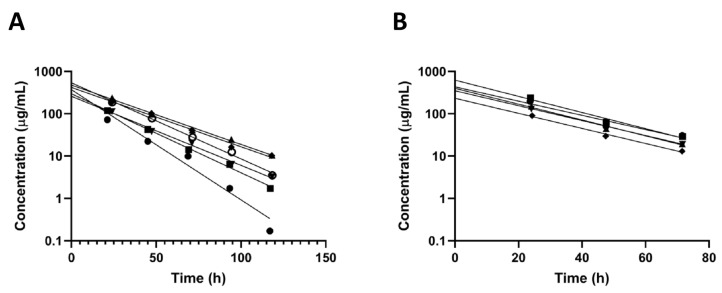
Concentrations of fluorescently labelled (**A**) BDP-pullulan (*n* = 6 eyes) and (**B**) BDP-pullulan-DEX (*n* = 5 eyes) in the vitreous of rats. One compartment model with first-order elimination rate constant was used for curve fitting (lines). The derived kinetic parameters are shown in Table 2. Each line represents the measurement from individual rat eye.

**Figure 4:**
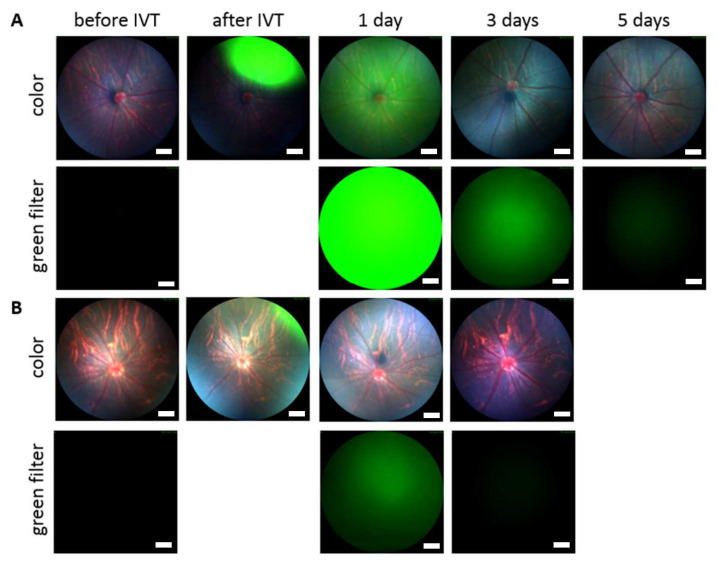
Full color and green fluorescent fundus images of rat eyes before and after intravitreal injection (IVT) of (**A**) BDP-pullulan and (**B**) BDP-pullulan-DEX. After one day, the labeled compounds distribute homogeneously in the vitreous. The length of scale bar is 200 µm.

Intravitreally administered BDP-pullulan and BDP-pullulan-DEX were monitored by fluorophotometry, fundus camera and OCT in rats. Vitreal elimination of pullulan formulations followed first-order elimination kinetics and the average vitreal half-life of both formulations was about 17 h in the rat eyes (Figure 3, Table 2). Fundus images showed that the formulations retained for about 3–5 days in the rat vitreous (Figure 4). The apparent volumes of distribution of pullulan conjugates (range of 42–84 µL) were close to the anatomical volume of rat vitreous (≈50 μL), whereas the vitreal clearance values (range of 1.8–3.5 μL/h) were lower than the average of aqueous humor flow rate in rats (21 μL/h) [48].

**Figure 5:**
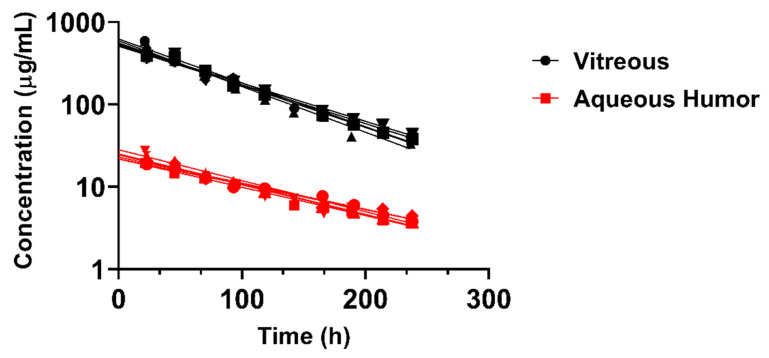
Concentrations of fluorescently labelled BDP-pullulan-DEX in the vitreous and aqueous humor of six rabbit eyes. Each line was fitted for the experimental data of one eye at different time points. One compartment model with first-order elimination rate constant was used for curve fitting.

BDP-pullulan-DEX concentrations in aqueous humor and vitreous were evaluated in rabbits by in vivo fluorophotometry. The vitreal half-life in rabbits (≈60 h) was longer than in the rats (≈17 h) and the elimination kinetics showed minimal inter-subject variability (Figure 5, Table 2). The average volume of distribution (932 μL) was close to the anatomical volume of vitreous humor in rabbits (≈1.5 mL) (Table 2). Moreover, in rabbits, the vitreal clearance (11 μL/h) was lower than the average of aqueous humor flow rate in normal albino rabbits (180 μL/h). The concentrations of BDP-pullulan-DEX in the aqueous humor were consistently about one order of magnitude lower than in the vitreous (Figure 5).

### 3.3. Pharmacokinetic Simulations

**Figure 6:**
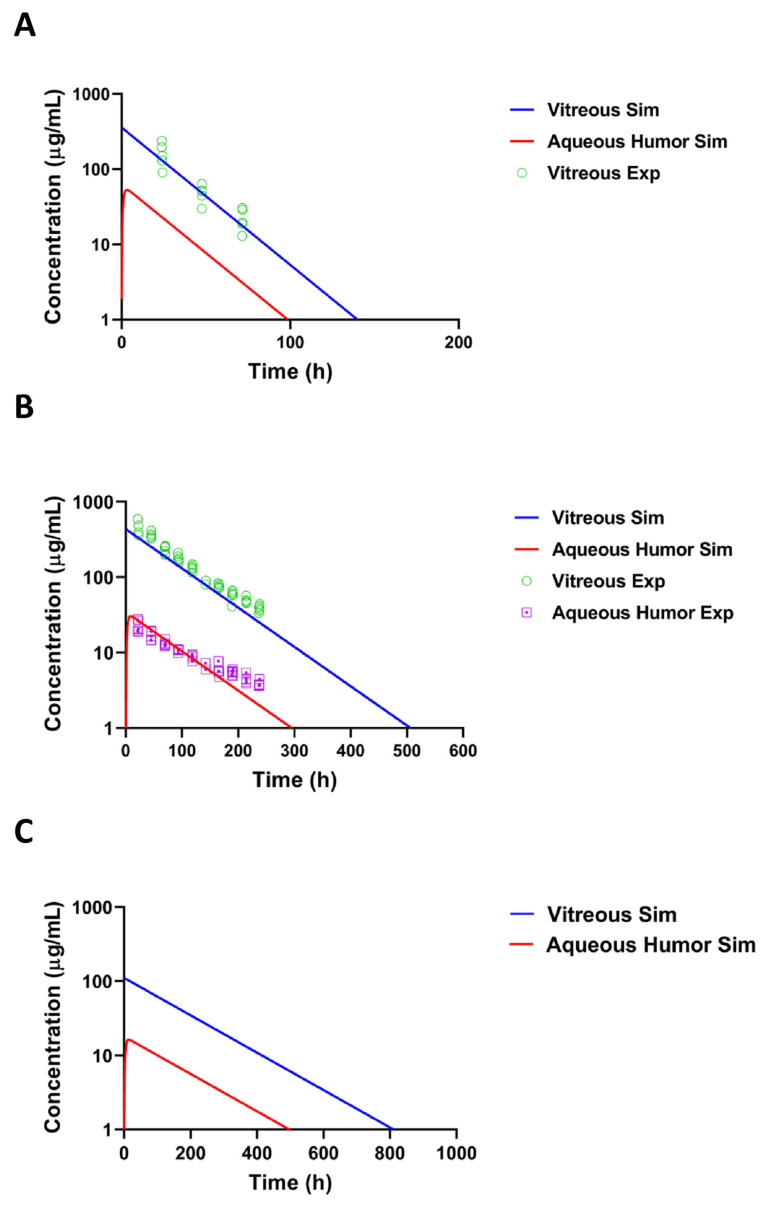
Simulated and experimental concentrations of BDP-pullulan-DEX in (**A**) rat, (**B**) rabbit and (**C**) human. The blue and red line shows the simulated concentration in vitreous and aqueous humor, respectively. The experimental concentrations in the vitreous and aqueous humor are indicated in the graphs.

A kinetic model (Figure 2) was used to simulate the concentrations of pullulan-DEX in the vitreous and aqueous humor. The simulated results matched the experimental results from rat and rabbit vitreous, when the model assumes that all elimination takes place anteriorly (Figure 6A,B). For rabbit eye, the simulated concentrations in the aqueous humor were remarkably close to experimental values, supporting the dominant role of anterior route for nanoparticle elimination from the vitreous. The experimental values for aqueous humor could not be measured accurately with fluorophotometry in the rat eyes. In order to scale the kinetics of the formulation from preclinical animals to human, the vitreal kinetics of pullulan conjugate were simulated in humans (Figure 6C). It is evident that the retention of pullulan conjugates is longer in the human eyes than in the rabbit eyes. Retention in the rabbit eyes is much longer than in the rat eyes (Figure 6A,B).

**Figure 7:**
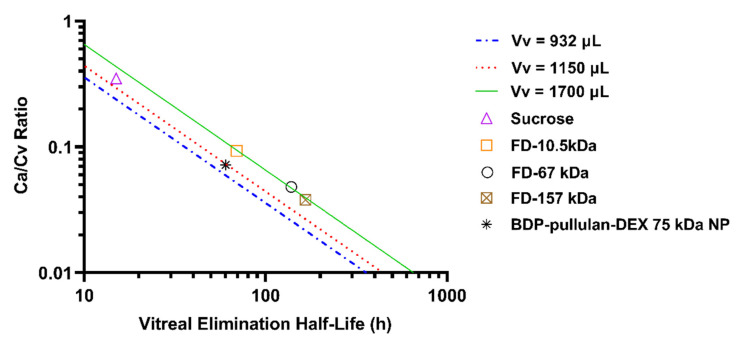
Maurice plot of intravitreally administered compounds in the rabbit eyes. The plot shows anteriorly eliminating compounds based on literature data: sucrose (0.342 kDa) [51], FITC-dextran (FD-10.5, FD-67 and FD-157 kDa) [52,53]. The green, red and blue line are derived from Maurice equation by assuming 932, 1150 and 1700 µL as vitreal volume of distribution. Location of BDP-pullulan-DEX NP (~75 kDa) data at close vicinity of the straight lines indicates anterior route of elimination in the rabbit eyes as the main elimination pathway.

The route of BDP labelled pullulan dexamethasone conjugate elimination in the rabbit eyes was further explored with Maurice plot that shows the relationship between C_a_/C_v_ and vitreal half-life (Figure 7) [49]. Location of pullulan conjugate in this plot, next to the straight line of compounds with anterior elimination route, supports the notion that the conjugate is mainly eliminated via anterior route. The plot is based on the equation C_a_/C_v_ = V_v_ k_v_/nf, where C_a_ is the concentration in the aqueous humor, C_v_ is the concentration in the vitreous humor, V_v_ is the volume of distribution in the vitreal compartment (unit: µL), k_v_ is the first order elimination rate constant (i.e., k_v_ = ln2/t_1/2_) (unit: h^−1^), f is the outflow rate of aqueous humor (unit: µL/min) and *n* is the coefficient that indicates the fraction of anterior drug elimination after intravitreal injection. The average experimental C_a_/C_v_ ratio is 0.072 ± 0.021. Using a literature value of f (3 µL/min) and experimental values for V_v_ (932 µL) and t_1/2_ (60.3 h), we obtained *n* value of 0.827 or 82.7% elimination via anterior route. At average rabbit V_v_ from the literature (1150 µL) [50], we obtained *n* value of 1.02 suggesting complete (≈100%) anterior elimination. Calculation based on both Vv (932 and 1150 µL) reveal anterior route as the main elimination pathway for pullulan conjugate. In Figure 7, three volumes of distribution (932, 1150 and 1700 µL) were used to derive the lines, indicating anterior chamber as the only elimination route.

**Figure 8:**
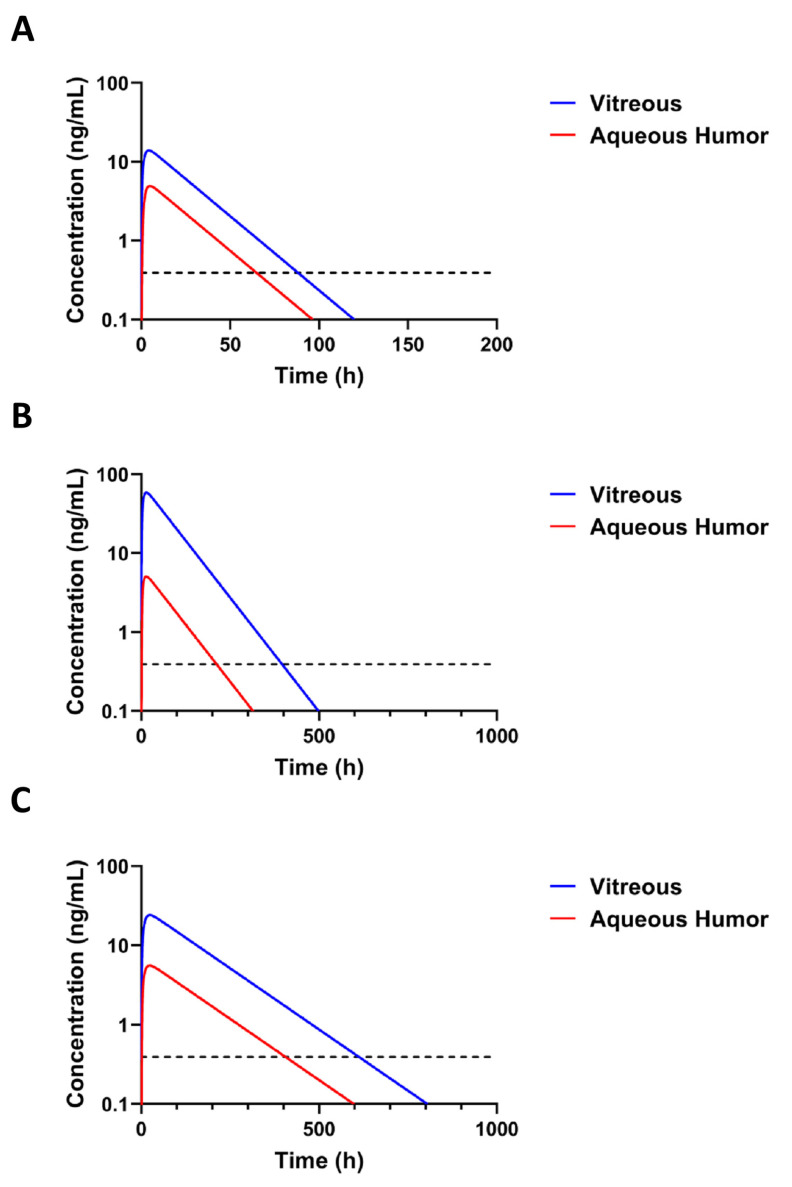
Simulation of released dexamethasone concentration in the vitreous and in the aqueous humor after intravitreal injection of BDP-pullulan-DEX in rat (**A**), rabbit (**B**) and human (**C**). The simulated doses of BDP-pullulan-DEX were 30, 500 and 500 µg per eye for rats, rabbits and humans, corresponding to DEX doses of 3, 50 and 50 µg per eye for rats, rabbits and humans, respectively. The dotted line (- - -) shows the minimum effective intravitreal concentration of dexamethasone for inhibiting the expression of VEGF, which is 1 nM (or 0.394 ng/mL) [54].

Concentrations of free dexamethasone were simulated based on the pullulan conjugate retention in the eye and the published [26] release rate of dexamethasone from dexamethasone-pullulan conjugates. The simulated levels of free dexamethasone after intravitreal injection of BDP-pullulan-DEX in rats and rabbits are presented in Figure 8A,B. The simulated dexamethasone concentrations in the vitreous remain above the minimal active concentration 1 nM (or 0.394 ng/mL) [54] for 3.6, 16.5 and 25.5 days in the rats, rabbits and humans, respectively (Figure 8A–C). The simulations also reveal that only a small fraction of injected dexamethasone in the conjugate is released during the residence time of the polymeric conjugate in the eye (Table 3). Interestingly, the concentrations in the vitreous and aqueous humor differ more in the rabbits and humans than in the rats.

### 3.4. Safety Assessment of Pullulan-Based Formulations

The safety of pullulan-based formulations was investigated ex vivo in mouse retinal explants and in vivo treated animals.

#### 3.4.1. Safety on Ex Vivo Mouse Retinal Explants

**Figure 9:**
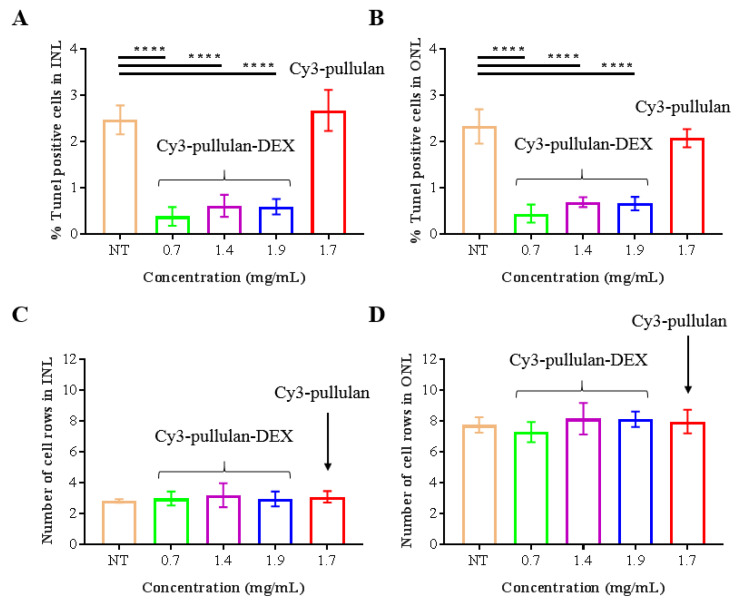
Ex vivo retinal explants of mice were treated with 15 μL of fluorescently labelled Cy3-pullulan-DEX (0.7, 1.4 and 1.9 mg/mL) and Cy3-pullulan (1.7 mg/mL). Untreated retina (NT) was used as control explant. TUNEL-positive nuclei in (**A**) the inner nuclear cell layer (INL), and (**B**) the outer nuclear cell layer (ONL) were counted and plotted as percentage of all nuclei in the INL and ONL areas. The number of cell rows in (**C**) INL and (**D**) ONL are also presented. Bars indicate standard deviations of means. One-way ANOVA **** *p* < 0.0001.

Ex vivo mouse retinal explants were treated with Cy3-pullulan (1.7 mg/mL) or Cy3-pullulan-DEX (0.7, 1.4 and 1.9 mg/mL) and untreated explants were used as controls. All three Cy3-pullulan-DEX concentrations significantly reduced the percentage of TUNEL positive cells in both the inner nuclear layer (INL) and the outer nuclear layer (ONL), indicating a reduction in the cell death in both layers (Figure 9A,B). This is due to the neuroprotective effect of DEX in ex vivo mouse retinal explants [55]. Treatment with Cy3-pullulan alone did not increase TUNEL labelled cells, indicating that the polymer had no toxic effects. The number of INL and ONL cell rows did not change (Figure 9C,D).

**Figure 10:**
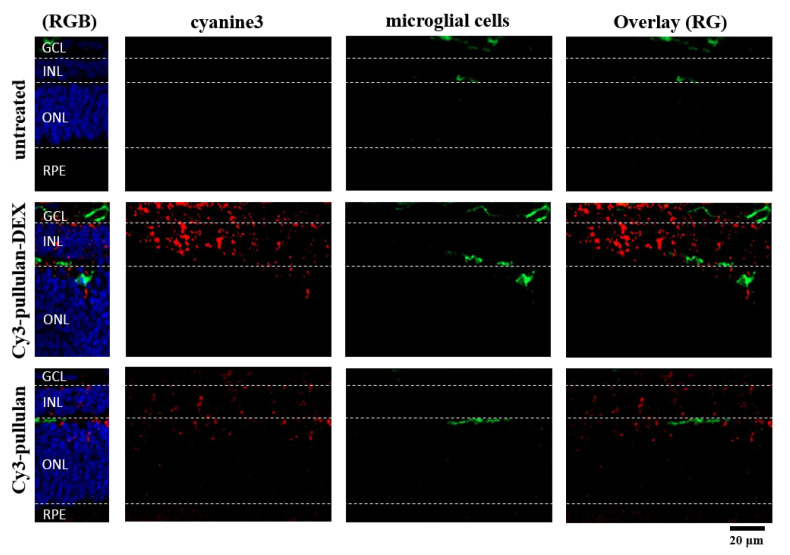
Microglial cells in sections of the ex vivo mouse retina labelled with an antibody against Iba-1 (green). Untreated retina, retina treated with Cy3-pullulan-DEX (15 μL, 1.9 mg/mL) and Cy3-pullulan (15 μL, 1.7 mg/mL) are shown. Cy3 fluorescence is shown as red. Nuclei were stained with DAPI (blue). RG: overlay of red and green channels for microglial cell, nanoparticle or conjugate colocalization. RGB: overlay of red, green and blue channels. Bar size: 20 μm.

To evaluate any inflammatory response due to the conjugates, we visualized the microglial cell shape and distribution using Iba-1 antibody, an inflammatory marker for microglia. The microglial cells were observed in ganglion cell layer (GCL) and INL in all groups, but no microglial migration to the ONL was seen (Figure 10). The conjugates (Cy3-pullulan, Cy3-pullulan-DEX) were found in all monitored layers, mostly in GCL and INL, and partially in ONL. The inflammatory response of microglia was not activated, and the conjugates are well tolerated in the ex vivo retinas.

#### 3.4.2. In Vivo Safety in Mice

In vivo safety of intravitreally injected pullulan formulations was evaluated in mice. Healthy status of the vitreous, retina and optic nerve were confirmed by fundus and OCT images 24 h after administration of fluorescently labelled Cy3-pullulan and Cy3-pullulan-DEX (SI-5, Figure S6). The animals were sacrificed, and the eyes were prepared for the TUNEL assay analysis that showed normal ONL and INL and no activation of cell death (SI-5, Figure S7).

#### 3.4.3. In Vivo Safety in Rats

**Figure 11:**
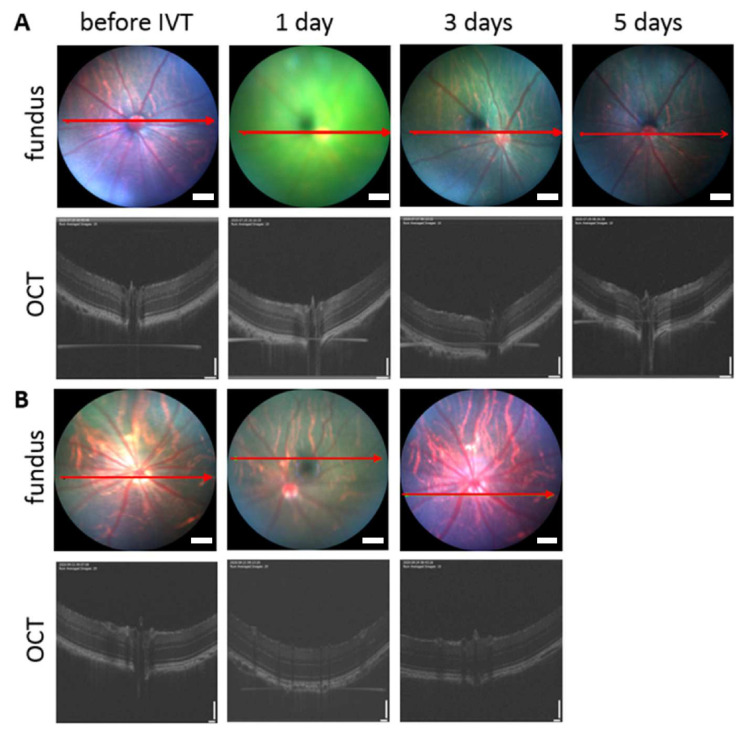
Fundus and optical coherence tomography (OCT) images of rat vitreous and retina. The figures are before and after intravitreal injections (1, 3 and 5 days) of BDP-pullulan (**A**) and BDP-pullulan-DEX (**B**). The length of scale bar for fundus images are 200 µm. In the case of OCT, vertical and horizontal scale bars in OCT are 110 and 130 µm, respectively.

In vivo safety of intravitreally injected pullulan formulations was evaluated in rats. Healthy status of the vitreous, retina, and optic nerve was confirmed by fundus and OCT images after exposure to BDP-pullulan and BDP-pullulan-DEX the following 3–5 days (Figure 11). Two weeks after intravitreal injections to the rat eyes, no visible alterations were seen (i.e., no conjunctival bleeding, cataracts, retinal detachment, swelling, clouding, changes in retinal morphology, cell debris or aggregation in the vitreous). No visual disturbance was detected since the vitreal clarity was confirmed by fundus imaging.

### 3.5. Retinal Penetration and Distribution

#### 3.5.1. Ex Vivo Studies

**Figure 12:**
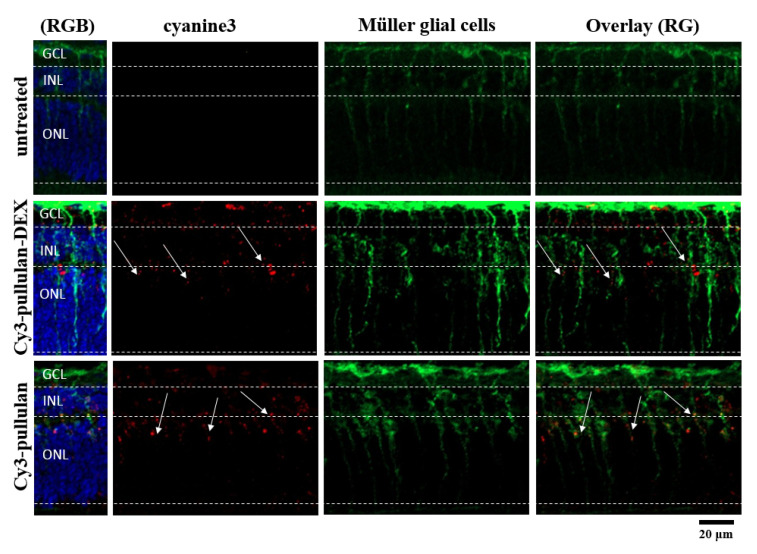
Müller glial cells in sections of the ex vivo mouse retina labelled with an antibody against glutamine synthetase (green). Untreated retina, retina treated with Cy3-pullulan-DEX (15 μL, 1.9 mg/mL) and Cy3-pullulan (15 μL, 1.7 mg/mL) are shown. Cy3 fluorescence is shown as red. Nuclei were stained with DAPI (blue). RG: overlay of red and green channels for Müller glial cell, nanoparticle or conjugate colocalization. RGB: overlay of red, green and blue channels. Bar size: 20 μm.

Distribution of pullulan-based formulations was studied in ex vivo mouse retinal explants (Figure 10 and Figure 12). The conjugates, Cy3-pullulan and Cy3-pullulan-DEX, were present in GCL, INL and ONL layers. The formulations were visualized in radial sections (red punctas, Figure 10 and Figure 12) through retinal layers overlapping with the Müller cells (Figure 12). Interestingly, the pullulan-based materials followed the route of Müller glial cells from the GCL to the distal part of the ONL, close to the retinal pigment epithelium (RPE) (Figure 12, 3D visualization in Supplementary Materials). Thus, this finding suggests that the pullulan-based conjugates may bypass the inner limiting membrane (ILM) via Müller cell phagocytosis in mouse retinal explants.

**Figure 13:**
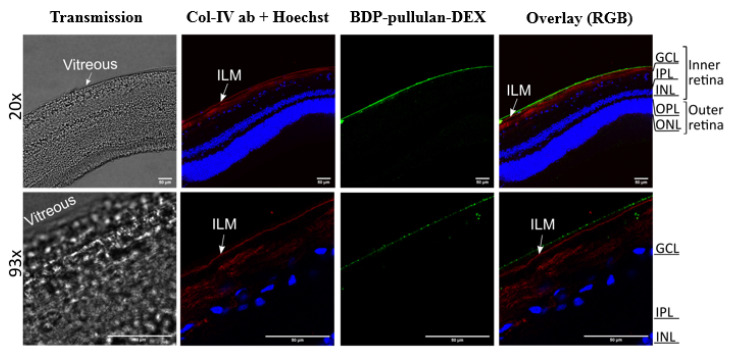
Retinal distribution of BDP-pullulan-DEX (100 μL, 5 mg/mL) in the vitreo-retinal ex vivo bovine explant 24 h after intravitreal injection. Representative confocal microscopy images of cryosections display the penetration of BDP-pullulan-DEX in the retinal layers (green). The inner limiting membrane (ILM) was labelled with rabbit anti-collagen type IV antibody (Col-IV ab, red). The vitreous can be seen as transparent layer in transmission imaging which is well aligned along the ILM while it appears in bright green color due to the high load of BDP-pullulan-DEX in merged channel mode. Nuclei are stained with Hoechst (blue). The white bar on the right bottom corner of each picture indicates the bar size: 50 μm. Abbreviations indicated in image: ganglion cell layer (GCL), inner plexiform layer (IPL), inner nuclear layer (INL), outer plexiform layer (OPL), outer nuclear layer (ONL), overlay of red/green/blue channels (RGB).

The retinal distribution of pullulan conjugates was also studied in ex vivo bovine retinal explants. In this tissue model, the nanoparticles were mainly localized at ILM. However, several sections showed a few signals (green punctas) from BDP-pullulan-DEX in GCL of the retina (Figure 13).

#### 3.5.2. In Vivo Mouse Experiments

**Figure 14:**
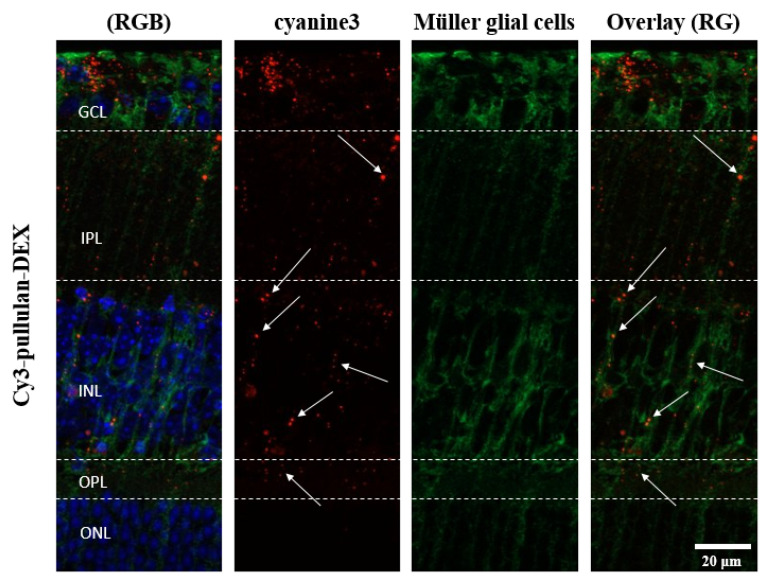
Images of retinal sections from two-months old mice after intravitreal injection (1 μL) of 5 mg/mL Cy3-pullulan-DEX (cyanine3, red). Müller glial cells were labelled with an antibody against glutamine synthetase (green). Nuclei were stained with DAPI (blue). RG: overlay of red and green channels for Müller glial cell and nanoparticle colocalization. RGB: overlay of red, green and blue channels. Bar size: 20 μm.

Two-months old mice were injected intravitreally with Cy3-pullulan-DEX. The results show localization of injected material across the retina, from inner to outer nuclear layers, mostly following Müller glial cells (Figure 14). This is in line with the ex vivo observations (Figure 12). Many particles accumulated in the cell bodies of Müller glial cells in the inner nuclear layer, but this was not seen in the other parts of the retina.

In adult mice, the presence of ILM in vivo and the penetration of pullulan material was detected as red dots in the Apotome microscopic images through GCL, INL and slightly in ONL layers (SI-5, Figures S8 and S9). The red fluorescently labelled pullulan samples were detected mostly in the GCL, inner plexiform layer (IPL) and INL layers and few dots were slightly visible in outer plexiform layer (OPL) and ONL (Figure 14).

## 4. Discussion

*Elimination kinetics*. The intravitreal pharmacokinetics and safety of pullulan–dexamethasone conjugates were investigated in detail. The results indicate that the conjugates are safe after intravitreal injections to the rodent eyes. Furthermore, they show prolonged retention in the rabbit eyes and retinal distribution via Müller glia cells in mouse eyes.

Non-modified pullulan behaves as a random coil of individual polymeric chains with diameters of few nanometers [13,33]. In this study, we used conjugates of pullulan with hydrophobic compound DEX and BDP. Pullulan conjugate with BDP was used since it allows direct and non-invasive monitoring of the particle kinetics after intravitreal injections. A roughly two-fold size difference was seen between pullulan-DEX and BDP-pullulan-DEX, but this should not cause kinetic differences since even several fold changes in liposome diameter (from <50 nm to >200 nm) did not change diffusivity in the vitreous [56]. These conjugates self-assemble to nanoparticles that showed similar retention times (half-lives ≈2.5–6.0 days) in the rabbit vitreous as macromolecules (e.g., antibodies and FITC-dextrans) [15], but shorter than the retention of some polymeric micelles and polymersomes (half-lives ≈9–31 days) [12]. Based on particle size, longer half-lives would be expected for pullulan conjugates, but the pullulan conjugates are structurally different systems than polymeric micelles and polymersomes. Furthermore, pullulan conjugates showed even distribution in the rat vitreous already at one day after injection, suggesting fast diffusion of the pullulan conjugates in the rat vitreous. The reasons for the described kinetic profile of pullulan conjugates are not known, but could involve biological interactions or particle disassembly. Disassembly of the particles to individual polymer conjugates might explain the results, but particle exposure to homogenized vitreous did not indicate particle disassembly in our previous study [26]. In vivo studies of nanoparticle disassembly and other mechanisms in the vitreous are methodologically challenging and the molecular mechanisms of pullulan-DEX elimination remain open for the time being.

In order to elucidate the elimination route of pullulan conjugates from the vitreal cavity, we performed kinetic simulations by assuming that all polymer is eliminated to the anterior chamber at the experimental elimination rate from the vitreous and further eliminated from the anterior chamber at the rate of aqueous humor outflow. The simulated particle concentrations in the aqueous humor were close to the experimental values, indicating that practically all pullulan conjugates are eliminated from the vitreous via anterior route. Moreover, the Maurice plot (C_v_/C_a_ vs. vitreal half-life) derived estimates of anterior elimination suggested that the major fraction (at least 82.7%) of intravitreally injected pullulan conjugate is eliminated via anterior route [49]. An anterior elimination route has been shown earlier for soluble macromolecules (e.g., antibodies) [57,58], but we have shown here for the first time that this route dominates the vitreal elimination of nanoparticles.

According to the observed kinetics of pullulan-DEX in rats and rabbits, the vitreal elimination half-life was longer in the rabbits. This is in line with our previous data with FITC-dextrans in rats and rabbits [15]. The current data shows that similar species difference is seen also with nanoparticles. A bigger size of the rabbit eye explains the longer half-life in rabbits, since it takes longer time for material transfer from the vitreous to the anterior chamber [58]. In humans, the volume of vitreous is about three times (≈4.5 mL) bigger than in rabbits (≈1.5 mL) [59], leading to even longer retention. Possible effects of inflammation on the vitreal kinetics of pullulan conjugates are not known. Based on the literature it is not likely that clearance of released dexamethasone would change due to inflammation [60]. But infiltrated macrophages due to ocular inflammation [61] might take up the particles [62], leading to faster drug release in the lysosomal conditions in the cells.

*Interplay of polymer conjugate retention and drug release*. In this study, we used dexamethasone conjugate of pullulan, but did not measure concentrations of free pharmacologically active dexamethasone in the vitreous. We simulated free dexamethasone concentrations in the vitreous and aqueous humor before designing and performing labor intensive, expensive and analytically demanding in vivo studies to determine free and bound dexamethasone concentrations in the eye. Kinetic simulations were based on experimental kinetics of pullulan conjugates and in vitro release of dexamethasone from the conjugates [26]. For the first time in scientific literature, these simulations integrate the drug release rate and pharmacokinetics of particulate intravitreal drug delivery system. The results demonstrate that longer effective residence of dexamethasone in the rabbit vitreous (up to 16.5 days in rabbits and 25.5 days in humans) is clearly longer than the half-life of the delivery system (3.6 days) in the rabbit vitreous. This is possible because the residual nanoparticles still release dexamethasone that is active at small concentrations. At the threshold limit of dexamethasone activity (0.394 ng/mL) [54], the simulated amount of free drug in the eye is in the range of 1–4 ng in rabbit and human eyes. This is only less than 0.001% of the injected dexamethasone dose within the pullulan conjugate. Overall, this demonstrates the power of controlled release technology in intravitreal drug delivery and possibilities for further optimization.

The minimum effective dexamethasone concentration may be different at different pathological conditions. Here we chose 1 nM as the minimum therapeutic concentration [54]. This concentration of dexamethasone should inhibit expression of vascular endothelial growth factor in the human vascular smooth muscle cultured cells. The concentration of endogenous cortisol is 14 nM in the human vitreous [63]. Since the anti-inflammatory potency of dexamethasone is about 25 times higher than that of cortisol [64], dexamethasone should be anti-inflammatory at 1 nM concentration. After administration of dexamethasone implant (dose 700 µg) to the vitreous of rhesus monkeys, the levels of dexamethasone remained above 1 nM for 3 months, but the efficacy may be extended even at lower levels of dexamethasone [65]. Thus, efficacy of pullulan conjugates may extend beyond 25 days as reported here, and this technology may be further developed for more extended ocular retention.

Interestingly, our simulations on retention and release revealed that a major fraction of the conjugated dexamethasone dose will be eliminated from the eye as pullulan conjugate form (>99%), yet the drug action may be significantly prolonged. Ocular exposure to the released drug can be defined as intraocular bioavailability. Intraocular bioavailability of the released drug could be improved by increasing drug release rate (e.g., modification of chemical linker) and prolonging polymer-conjugate retention in the vitreous (e.g., increasing binding with vitreal components and molecular weight of polymer). The release and retention profiles of pullulan conjugated dexamethasone are very different from the intravitreal dexamethasone implants (e.g., Retisert). The implants release all the drug payload in the eye and thereafter a ‘ghost matrix’ remains in the eye. Interplay of delivery system retention, drug release and ocular pharmacokinetics has not been discussed in the literature. The presented simulation models provide useful tools for drug developers who optimize intravitreal drug delivery systems towards target product profiles.

*Retinal permeation*. The inner limiting membrane (ILM) is a mechanical and electrostatic barrier that limits access of nanoparticles into the retina. The ILM includes a negatively charged network of collagen and glycosaminoglycans [66]. Retinal delivery of materials with the molecular weights ≤ 100 kDa are not limited by ILM [67]. However, the electric charge of the materials can make a difference: FITC-dextrans of 20 kDa and 500 kDa permeated through ILM, but cationic poly-L-lysine (20 kDa) did not [68]. Recently, liposomes (≈50 nm) with negative charge and PEG-coating were shown to permeate across bovine ILM that is considered a close model for human ILM [44]. However, larger liposomes of about 100 nm in diameter were not able to cross the ILM barrier in bovine retina. In this study, pullulan conjugates (particle size 200–400 nm) were permeating only to the ganglion cell layer of the bovine ex vivo retina, but not further into the retina. Ganglion cells are an important drug target, especially in the treatment of glaucomatous retinal degeneration [69]. Limited ILM permeation is also an obstacle in the field of retinal gene therapy with viral vectors and, for this reason, retinal gene therapy is performed using subretinal injections [70]. Better understanding of the ILM and other contributing factors will help in development of intravitreal treatments of retinal diseases. In various retinal disorders and upon ageing, ILM may become thinner and presents pores thereby become leakier to the nanoparticles [60].

Retinal permeation of pullulan was also investigated with organotypic mouse retinal explant that can be maintained as biologically active even for one month [71]. This model is useful in mechanistic pharmacological studies [1,2,72,73,74,75]. ILM in mouse retina is thinner and leakier than in the bigger eyes, potentially giving optimistic views on retinal permeation of particles [44]. However, this study showed qualitatively distinct retinal distribution: pullulan conjugates did not cross ILM, but they were taken up by the Müller cells that may not be as active in the isolated bovine retina. The pullulan–dexamethasone nanoparticles showed protective effects in the retina, suggesting drug release from the conjugates in the retina.

Delivery of pullulan conjugates to Müller cells can be explained on the location and properties of these cells. Müller cells are glial cells capable of phagocytosing cell bodies, fragments of retinal cells and particles [76,77,78]. The cell bodies of Müller glia are located in the inner nuclear layer with its two stem processes extending in opposite directions and spanning the entire retina. Therefore, it is likely that intravitreal particles could be phagocytosed by Müller cells and carried into the inner retinal layers. In this study, the cultured retinal explants from wild-type mice were exposed to pullulan-based particles from the ganglion cell layer side on the retina. Colocalization with glutamine synthase antibody (Müller glia marker) confirmed particle localization in Müller cells and the particles were visualized in radial sections (red punctas, Figure 12) through all retinal layers in a linear distribution similar to the Müller cells extension (from the ganglion cell layer until reaching the distal part of the outer nuclear layer (Figure 12, and 3D visualization in Supplementary Materials). This indicates that the pullulan-based particles were phagocytized by the Müller cells, thereby overcoming the barrier of inner limiting membrane.

Müller glial cells are a major type of macroglial cells in the neural retina [79,80,81,82,83]. The close interactions that Müller cells have with other cells are crucial in the actions of antioxidants, neurotrophic factors and growth factors in the retina [80]. In this respect, Müller cells are an important cell target in retinal drug development and targeted localization of pullulan conjugates to the Müller cells opens possibilities to deliver drugs to these cells in a targeted manner. Furthermore, biocompatibility of the materials was confirmed in more detail in ex vivo studies with mouse organotypic retinal explants.

*Pullulan conjugates as potential intravitreal drug delivery systems*. The preclinical results showed that pullulan is a safe polymeric backbone for intravitreal drug delivery and capable of extending dexamethasone retention in the eye.

Previously, polymeric drug conjugates have been widely investigated as drug delivery systems for cancer treatment [84,85,86]. For ocular treatments, polymeric drug conjugates have been only sparsely studied [59,66,67]. In principle, polymeric conjugates provide major advantages: (1) large molecular size prevents elimination across blood–ocular barriers and slows diffusion to the anterior chamber, thereby prolonging drug residence in the eye; (2) covalent conjugation of drugs to the polymer backbone enables controlled drug release from soluble free polymer conjugates and self-assembled nanoparticles. Control of drug release is particularly significant because non-covalent drug loading to nanosystems results often in relatively fast drug release. Furthermore, covalent links allow generation of site-specific drug release in retina [10] or even in sub-cellular compartments [87].

Furthermore, OCT and fundus imaging showed no opacity in the vitreous after intravitreal pullulan conjugate injection, representing another advantage compared to implants, suspensions and microspheres [88]. Dexamethasone could be delivered as a water solution of polymeric conjugate without any visual disturbance. Further, the conjugated drug would not permeate to the lens, thereby decreasing the risk of corticosteroid cataract. Long-term exposure of dexamethasone may also cause higher intraocular pressure through increasing the stiffness of the trabecular meshwork [89]. Low dexamethasone dose (50 µg) and only partial release (<2.5 µg) from pullulan-DEX (50 µg) within the eye should reduce free drug exposure to the lens and trabecular meshwork to negligible levels as compared to the exposure after intravitreal administration of Ozurdex implant (700 µg) and dexamethasone suspension (50 µg). This factor may reduce the incidence of ocular adverse effects. The small needle size is another potential advantage of intravitreal polymeric conjugates. Compared to the 22 G Ozurdex needle the required needle size (31–32 G) for polymer conjugate is minimal thereby reducing the invasiveness of the treatment.

## 5. Conclusions

Delivery of intravitreally administered drugs may be improved with innovative drug delivery systems enabling more patient compliant administration, prolonged drug retention in the eye, controlled release and delivery to the retinal target cells. This research work demonstrates that dexamethasone conjugates of pullulan have such features. Pullulan–dexamethasone nanoparticles were safe in the preclinical animal models, distributed to the ganglion cells and Müller cells and showed prolonged ocular retention. Pharmacokinetic models and simulations demonstrated important aspects in the interplay of ocular retention and drug release. These models will be useful tools in the field of ocular drug delivery system development. Overall, pullulan-based drug conjugates are a promising drug carrier platform for the intravitreal drug delivery.

## Figures and Tables

**Table 1:** Intensity based mean sizes (±SD), polydispersity indices (PDI) and zeta potentials of the pullulan conjugate nanoparticles. The measurements were performed by dynamic light scattering.

Sample	Mean Size ± SD (nm)	PDI	Zeta Potential (mV)
pullulan-DEX	461 ± 30	0.39 ± 0.04	−38.1 ± 0.5
Cy3-pullulan-DEX	299 ± 42	0.22 ± 0.11	−20.3 ± 3.0
BDP-pullulan-DEX	219 ± 15	0.25 ± 0.07	−40.9 ± 1.1

**Table 2:** Kinetic parameters of intravitreally injected fluorescently labelled: BDP-pullulan (*n* = 6 rat eyes) and BDP-pullulan-DEX (*n* = 5 rat eyes; *n* = 6 rabbit eyes) derived from fluorophotometric measurements.

Material	Dose (μg)	Species	C_0_ (μg/mL)	t_1/2_ (h)	V_d_ (μL)	CL (μL/h)
BDP-pullulan	15	rats	386.4 ± 110.2	17.4 ± 3.9	42 ± 12	1.8 ± 0.7
BDP-pullulan-DEX	30	rats	393.7 ± 134.1	16.7 ± 0.8	84 ± 30	3.5 ± 1.2
BDP-pullulan-DEX *	500	rabbits	539.3 ± 43.1	60.3 ± 4.9	932 ± 72	11 ± 0.4

* The elimination half-life in rabbit aqueous humor was 87.5 ± 7.3 h.

**Table 3:** Pharmacokinetic parameters of dexamethasone derived from simulations of intravitreally injected BDP-pullulan-DEX to rat, rabbit and human eyes. The dose of dexamethasone was 3, 50 and 50 µg per eye for rats, rabbits and humans, respectively.

Parameter	Unit	Rat	Rabbit	Human
C_max Vitreous_	ng·mL^−1^	14	58	24
T_max Vitreous_	h	5	15	24
C_max Aqueous Humor_	ng·mL^−1^	5	5	6
T_max Aqueous Humor_	h	5	15	24
Aqueous humor/vitreous concentration ratio ^a^		0.35	0.086	0.23
Duration above minimal effective concentration	day	3.6	16.5	25.5
Dose of BDP-pullulan-DEX per eye	μg	30	500	500
Percent of the released DEX in the vitreous	%	0.8	2.5	4.7
Percent of the released DEX in the aqueous humor	%	0.028	0.05	0.04

^a^ Averaged at pseudo-steady state phase.

## Data Availability

Data are contained within the article and in Supplementary Materials. The additional data presented in this study are available on request from the authors.

## Supplementary Materials

The following are available online at https://www.mdpi.com/article/10.3390/pharmaceutics14010012/s1. SI-1 and Scheme S1: Synthesis of pullulan-DEX conjugates; SI-2 and Figure S1: Ex vivo mouse retinal organ culture; SI-3 and Figures S2 and S3: Calibration curve for in vivo fluorophotometry measurements; SI-4, Figures S4 and S5 and Table S1: Pharmacokinetics simulations; SI-5 and Figures S6–S9: Safety on in vivo mice model; 3D visualization available online.
