# Supplementary material for: Pharmacokinetics of Pullulan–Dexamethasone Conjugates in Retinal Drug Delivery
Source: Pharmaceutics. 2021 Dec 21;14(1):12. doi: 10.3390/pharmaceutics14010012 (PMC8779473; doi:10.3390/pharmaceutics14010012)
Supplement: Supplementary file 1 [file pharmaceutics-14-00012-s001.zip › pharmaceutics-1498513-supplementary.pdf]

Supplementary Materials: Pharmacokinetics of Pullulan–Dexamethasone Conjugates in Retinal Drug Delivery

Eva Kicková, Amir Sadeghi, Jooseppi Puranen, Shirin Tavakoli, Merve Sen, Veli-Pekka Ranta, Blanca Arango-Gonzalez, Sylvia Bolz, Marius Ueffing, Stefano Salmaso, Paolo Caliceti, Elisa Toropainen, Marika Ruponen and Arto Urtti

SI-1 Synthesis of pullulan-DEX conjugates

The synthesis of pullulan-DEX conjugates (Scheme S1) was based on a multistep procedure described previously in detail [1]. Briefly, in Scheme S1, I.) pullulan was activated through carboxyethyl-pullulan, II.) converted to carboxyhydrazide-pullulan, and then III.) conjugated with DEX and IV.) fluorescent labels Cy3 or BDP (general name: 'label' in Scheme S1). Pullulan-DEX was obtained with 90% mol/mol recovery yield; 5.2% GPU (glucose per unit) DEX group derivatization yield corresponding to 10% w/w conjugation yield of DEX.

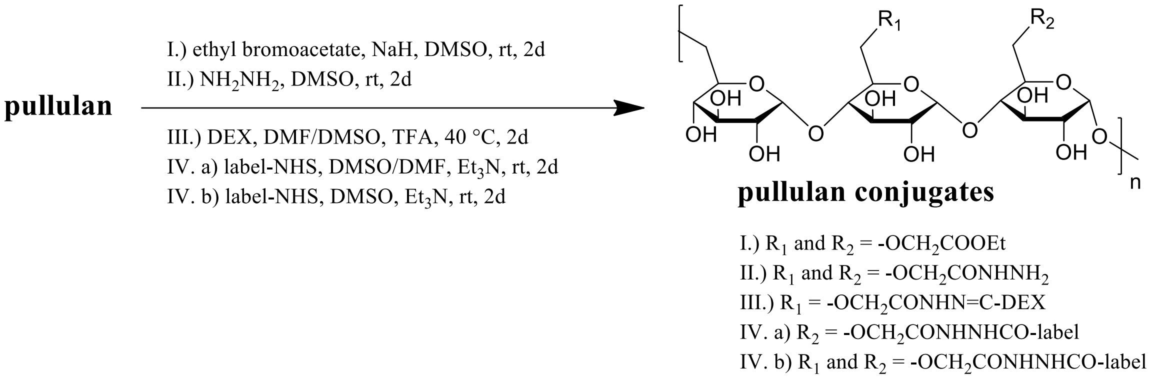

Scheme S1. Schematic representation of pullulan–dexamethasone conjugate synthesis. Fluorescent labels Cy3 or BDP have the general name: 'label' in Scheme. Details can be found in our recent publication [1].

SI-1.1. Synthesis of BDP-pullulan-DEX

Pullulan-DEX labelling with BDP was performed according to published protocols [1,2]. Briefly, pullulan-DEX (0.19 g, 0.9 mmol GPU) was dissolved in 62 mL of 4:1 *v/v* DMSO/DMF anhydrous mixture followed by triethylamine (TEA, 0.01 mL, 0.07 mmol) and maintained at room temperature for 2 hours. BDP-FL-NHS ester (0.012 g, 0.03 mmol) dissolved in 1.1 mL of

anhydrous DMSO was added to the pullulan-DEX solution. The reaction was maintained under nitrogen atmosphere and stirring continued at room temperature for 2 days in the dark. The conjugate was isolated by precipitation and washed with DCM as reported before [1] to eliminate unconjugated BDP label. BDP-pullulan-DEX was obtained as orange powder with 89% mol/mol recovery yield and 5.2% GPU DEX derivatization yield and 1.1% GPU BDP derivatization yield.

^{1}H NMR (400 MHz, DMSO-d_6, with internal standard (IS) 4-chloro-3-nitrobenzoic acid): δ 8.34 (d, 1 aromatic proton (H*ar*), IS), 8.08 (dd, 1H*ar*, IS), 7.69 (d, 1H*ar*, IS), 5.62-3.00 [1H, (1→4)-α-glycosidic bond; 1H, (1→6)-α-glycosidic bond; 5H, remaining Hs of glucopyranose; 2H, -CH_2-, carboxyethyl group; 12H, remaining Hs of dexamethasone; 4H, 2x -CH_2-, bodipy], 2.67 (p, 3H, -CH_3, bodipy), 2.33 (p, 3H, -CH_3, bodipy), 1.24 (s, 3H, CH_3*ar*, dexamethasone), 1.11 (d, 3H, -C-CH_3, dexamethasone), 0.84 (d, 3H, -CH-CH_3, dexamethasone).

SI-1.2. Synthesis of BDP-pullulan

Carboxyhydrazide-pullulan (0.18 g, 1.01 mmol GPU) was dissolved in 10 mL of anhydrous DMSO. 0.12 mL of BDP-FL-NHS ester (0.012 g, 0.03 mmol) solution in anhydrous DMSO was added to the mixture under stirring in the dark followed by 0.01 mL TEA (0.07 mmol). The reaction mixture was stirred at room temperature for 2 days in the dark. The polymer was isolated by precipitation in DCM as described previously [1]. The orange solid BDP-pullulan was obtained with 86% mol/mol recovery yield and 2.2% GPU BDP derivatization yield.

^{1}H NMR (400 MHz, D_2O): δ 7.56 (s, 1H*ar*, bodipy), 7.16 (d, 1H*ar*, bodipy), 6.50 (d, 1H*ar*, bodipy), 6.40 (s, 1H*ar*, bodipy), 5.43 (d, 1H, (1→4)-α-glycosidic bond), 5.00 (s, 1H, (1→6)-α-glycosidic bond), 4.60-3.40 [5H, remaining Hs of glucopyranose; 2H, -CH_2-, carboxyethyl group; 4H, 2x -CH_2-, bodipy], 2.59 (s, 3H, -CH_3, bodipy), 2.34 (s, 3H, -CH_3, bodipy); ^{1}H NMR (400 MHz, DMSO-d_6, with internal standard (IS) 4-chloro-3-nitrobenzoic acid): δ 8.39 (s, 1H*ar*, IS), 8.11 (d, 1H*ar*, IS), 7.77 (d, 1H*ar*, IS), 6.53 (s, 1H*ar*, bodipy), 5.62-3.00 [1H, (1→4)-α-glycosidic bond; 1H, (1→6)-α-glycosidic bond; 5H, remaining Hs of glucopyranose; 2H, -CH_2-, carboxyethyl group; 4H, 2x -CH_2-, bodipy), 2.67 (s, 3H, -CH_3 bodipy), 2.33 (s, 3H, -CH_3 bodipy).

The similar labelling procedures were applied and published for Cy3-pullulan-DEX and Cy3-pullulan together with more details about the synthesis procedures, purification methods and characterizations [1].

SI-2 *Ex vivo* mouse retinal organ culture

The brief schematic interpretation of the preparation of organotypic retinal culture and maintenance of the retinal explants is placed in Figure S1. All procedures were performed according to published protocols [3,4]. Briefly, PN6 mice were sacrificed and the eyes were enucleated in an aseptic environment and pretreated with 12 % proteinase K (MP Biomedicals, 0219350490) for 15 minutes at 37 °C in Neurobasal® Medium (GIBCO, Thermo Fisher Scientific, Dreieich, Germany). The enzymatic digestion was stopped by the addition of 20 % FBS (Sigma–Aldrich, F7524). Retinae were isolated with the RPE attached and four radial cuts were made to flatten it as described previously [5,6]. Then, the tissue was transferred to a 0.4 μm polycarbonate

membrane (Corning Life Sciences, CLS3412), the RPE side touching the membrane. The inserts were placed into 6-well culture plates and incubated in complete medium (Neurobasal® Medium, B-27® Supplement, N-2 supplement, GlutaMAX™ from GIBCO, Thermo Fisher Scientific, Dreieich, Germany; antibiotics mixture containing 100 µg/mL Streptomycin and 100 units/mL Penicillin from PAN Biotech, Aidenbach, Germany) at 36.5 °C.

The tissues were randomly assigned to the following treatment groups with pullulan conjugates: Cy3-pullulan (1.7 mg/mL), Cy3-pullulan-DEX (0.7, 1.4, and 1.9 mg/mL) and untreated control. The treatments were carefully applied on the top of the retinal tissues (on the ganglion cell layer) using volumes of 15 µL. No treatment was applied on the control tissues. Six retinal tissues were used for each group. The complete medium was changed every 48 hours and maintained in a humidified atmosphere of 5% CO_2 at 37 °C for six days.

The tissues were immersed in 4 % paraformaldehyde in 0.1 M phosphate buffer (PB; pH 7.4) for 45 minutes at 4 °C, followed by cryoprotection in graded sucrose solutions (10 %, 20 % and 30 %) and embedded in cryomatrix (Tissue-Tek® O.C.T. Compound, Sakura® Finetek, VWR, 4583). Radial sections (14 µm thick) were collected, air-dried, and stored at -20 °C. The visualization of dying cells of the retinal explants in all evaluated groups was provided using TUNEL assay [7], with the *in situ* cell death detection kit conjugated with fluorescein isothiocyanate (Roche, 11684795910). DAPI (Vectashield Antifade Mounting Medium with DAPI; Vector Laboratories, H-1200) was used as a nuclear counterstain. Sections were incubated overnight at 4 °C with Iba-1 or glutamine synthetase specific antibodies. Fluorescence immunocytochemistry was performed using Alexa Fluor® 488 conjugated secondary antibody (Invitrogen, A-11031). Negative controls were carried out by omitting the primary antibody. The obtained images were then processed with ImageJ software 1.52n.

All samples were analyzed using a Zeiss Axio Imager Z1 ApoTome microscope, AxioCam MRm camera and Zeiss Zen 2.3 software in Z-stack at 20x magnification. For quantitative analysis, positive cells in the ONL of at least five sections per group were manually counted.

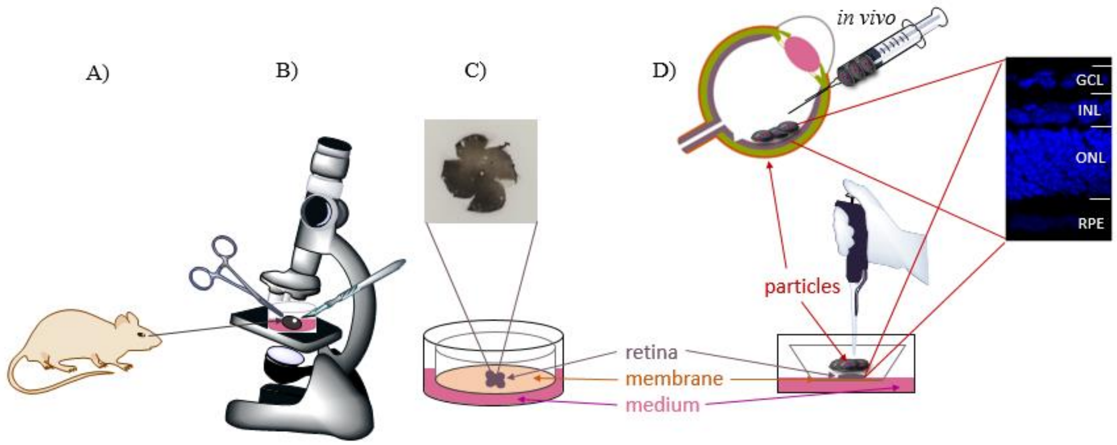

Figure S1. Schematic representation of the *ex vivo* retinal organ culture method: (**A**) six-day-old wild-type C57BL/6 mouse model; (**B**) removing the eyeball and preparation of the retina tissue under dissection microscope; (**C**) isolated retina tissue placed on the membrane insert with medium; and (**D**) application of the particle solution (pullulan-DEX or pullulan) on the top of the retina tissue and correlation with the *in vivo* intravitreal injection of particle solution with detailed representation of the cell layers in the retina (GLC, INL, ONL and RPE).

SI-3 Calibration curve for in vivo fluorophotometry measurements

The solution of BDP-pullulan was prepared in sterile PBS, pH 7.4 (5 mg/mL). Several dilutions (245, 175, 125, 75, etc. μg/mL) were prepared and measured by Fluorotron instrument to set up a calibration curve (Figure S2).

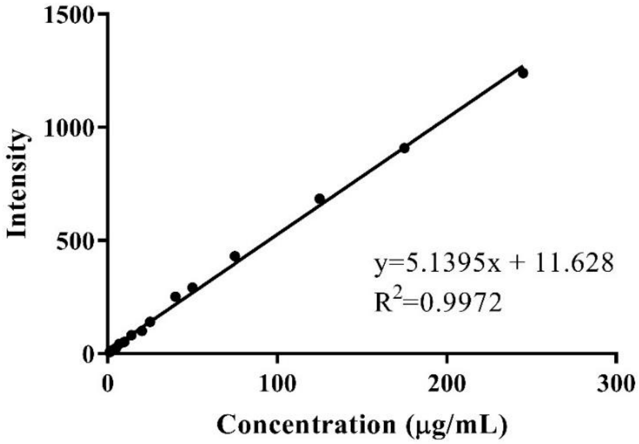

Figure S2. Calibration curve of BDP-pullulan measured by Fluorotron.

The solution of BDP-pullulan-DEX was prepared in sterile PBS, pH 7.4 (5 mg/mL). Several dilutions (500, 350, 250, 175, etc. μg/mL) were prepared and measured by Fluorotron instrument to set up a calibration curve (Figure S3).

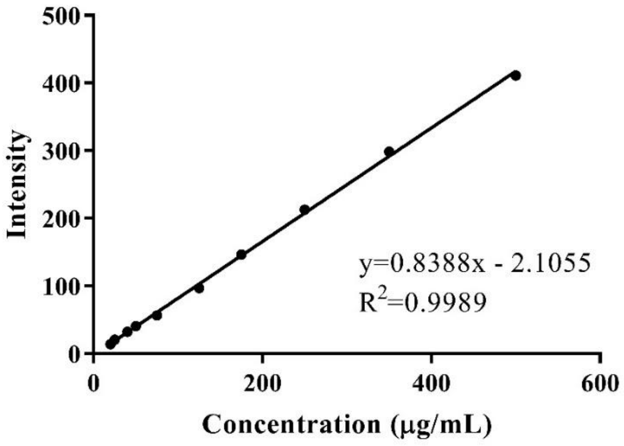

Figure S3. Calibration curve of BDP-pullulan-DEX measured by Fluorotron.

SI-4 Pharmacokinetics simulations

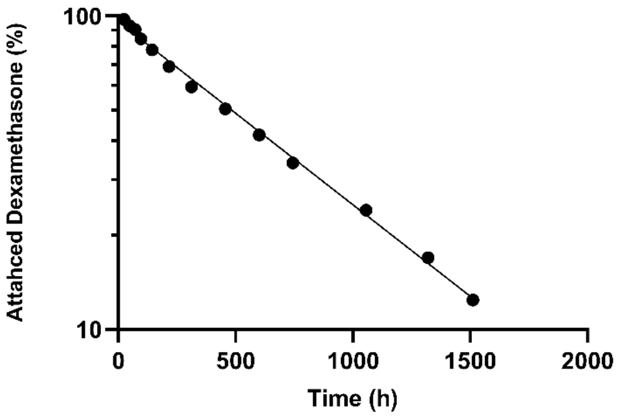

Figure S4. Percentage of releasable dexamethasone vs time. The maximum amount of releasable dexamethasone 125 μg from 5000 μg pullulan-DEX was considered as 100 percent [1]. The release data in the vitreous medium were used in this graph. The equation of fitted line is $Y = 95.88\ e^{-0.0013t}$. The weighting method of $1/Y^2$ was used for nonlinear fitting.

Table S1. Parameters used in kinetic simulations.

			Species		
Parameters in kinetic simulations	***Symbols***	***Units***	***Rat***	***Rabbit***	***Human***
**Vitreal elimination rate constant of pullulan-DEX**	K_{v-p}	h^{-1}	0.042 [a]	0.012 [a]	0.0058 [b]
**Vitreal volume of distribution**	V_v	µl	84 [c]	1150 [8]	4500 [9,10]
**Aqueous humor volume**	V_{AqH}	µl	18.2 [11]	287 [12]	250 [13]
**Vitreal elimination rate constant of dexamethasone**	K_{v-D}	h^{-1}	0.693 [d]	0.199 [14]	0.126 [15]
**Elimination rate constant of dexamethasone from anterior route**	K_{aqhD}	h^{-1}	1.5 [e]	1.5 [16,17]	1.5 [e]
**Elimination rate constant of dexamethasone from vitreous via posterior route**	K_{p-D}	h^{-1}	0.607 [f]	0.174 [f]	0.11 [f]
**Elimination rate constant of dexamethasone from vitreous via anterior route**	K_{p-D}	h^{-1}	0.086 [f]	0.025 [f]	0.016 [f]
**Intravitreal Injection volume**	V_{inj}	µl	3	50	100
**Intravitreal dose of pullulan-DEX**	D_P	µg	30 [g]	500 [g]	500 [g]
**Aqueous humor flow rate**	f	$µl.h^{-1}$	21 [11]	180 [18]	165 [19]
**Elimination rate constant of pullulan-DEX from the anterior chamber (f/VAqH)**	K_{aqhP}	$µl.h^{-1}$	1.15	0.627	0.66
**First order release rate constant of dexamethasone from pullulan-DEX**	$K_{r\_}$	h^{-1}	0.0013 [f]	0.0013 [f]	0.0013 [f]
**Ratio mass of releasable DEX to pullulan-DEX**	$M_{r-D/P}$	****	0.025 [f]	0.025 [f]	0.025 [f]
**Concentration of IVT injected solution of pullulan-DEX**	C_{inj}	$mg.ml^{-1}$	10	10	10
**Virtual delay rate constant for dexamethasone**	K_{dD}	h^{-1}	50 [i]	5 [h]	2.5 [i]
**Virtual delay rate constant for pullulan-DEX**	K_{dL}	h^{-1}	7 [j]	0.7 [k]	0.35 [j]

			Species		
Parameters in kinetic simulations	***Symbols***	***Units***	***Rat***	***Rabbit***	***Human***
**Vitreal elimination rate constant of pullulan-DEX**	K_{v-p}	h^{-1}	0.042 [a]	0.012 [a]	0.0058 [b]
**Vitreal volume of distribution**	V_v	µl	84 [c]	1150 [8]	4500 [9,10]
**Aqueous humor volume**	V_{AqH}	µl	18.2 [11]	287 [12]	250 [13]
**Vitreal elimination rate constant of dexamethasone**	K_{v-D}	h^{-1}	0.693 [d]	0.199 [14]	0.126 [15]
**Elimination rate constant of dexamethasone from anterior route**	K_{aqhD}	h^{-1}	1.5 [e]	1.5 [16,17]	1.5 [e]
**Elimination rate constant of dexamethasone from vitreous via posterior route**	K_{p-D}	h^{-1}	0.607 [f]	0.174 [f]	0.11 [f]
**Elimination rate constant of dexamethasone from vitreous via anterior route**	K_{p-D}	h^{-1}	0.086 [f]	0.025 [f]	0.016 [f]
**Intravitreal Injection volume**	V_{inj}	µl	3	50	100
**Intravitreal dose of pullulan-DEX**	D_P	µg	30 [g]	500 [g]	500 [g]
**Aqueous humor flow rate**	f	$µl.h^{-1}$	21 [11]	180 [18]	165 [19]
**Elimination rate constant of pullulan-DEX from the anterior chamber (f/VAqH)**	K_{aqhP}	$µl.h^{-1}$	1.15	0.627	0.66

**First order release rate constant of dexamethasone from pullulan-DEX**	$K_{r\_}$	**h^{-1}**	0.0013 [f]	0.0013 [f]	0.0013 [f]
**Ratio mass of releasable DEX to pullulan-DEX**	$M_{r-D/P}$	****	0.025 [f]	0.025 [f]	0.025 [f]
**Concentration of IVT injected solution of pullulan-DEX**	C_{inj}	**mg.ml^{-1}**	10	10	10
**Virtual delay rate constant for dexamethasone**	K_{dD}	**h^{-1}**	50 [i]	5 [h]	2.5 [i]
**Virtual delay rate constant for pullulan-DEX**	K_{dL}	**h^{-1}**	7 [j]	0.7 [k]	0.35 [j]

[a] The experimental values for vitreal elimination constant of pullulan-DEX are from the current study. [b] For estimating the elimination rate constant of pullulan-DEX in humans, the value of the rabbit was divided by two. The average time of the diffusion in the human is almost two times longer in humans compared to rabbits [20]; therefore, half of the values were assumed for humans. [c] Apparent volume of distribution for rats is from current study. This value is at the range of anatomical volume (~50 µl). In the case of humans, anatomical volume was assumed. The average volume of distribution after IVT injections was assumed in the case of rabbits, slightly lower than the anatomical volume (~1.5 ml) [21]. [d] The vitreal elimination rate constant of dexamethasone in rats is not yet known. The elimination rate constant of dexamethasone in rats was assumed to be similar to fluorescein. The elimination half-life of fluorescein in rats is about 1 hour. The half-life of fluorescein in rabbits is about 3 hours [22], which is within the same range of dexamethasone with 3.5 hours elimination half-life. Moreover, the molecular weight and lipophilicity of dexamethasone and fluorescein are in the same range ($logD_{7.4}$ for fluorescein and dexamethasone are 2.68 and 2.03, respectively[23]). [e] The elimination rate constant of the dexamethasone from the anterior chamber was considered to be similar for rats, rabbits and humans. [f] The contribution of the elimination rate from anterior route to the total vitreal elimination for dexamethasone was considered as 12.5 percent. In the case of rabbits, this value could reproduce the experimental values in the aqueous humor following IVT injection. This ratio is consistent with the average ratio of aqueous humor/vitreous to the vitreous surface if we consider that as an ideal sphere. As shown by Hutton-Smith et al. [13], this ratio is almost in the same range in different species, including rabbits, humans and monkeys. For rats and rabbits, the dose of conjugate was the same as the experimental dose in this study. In the case of humans, the same dose as rabbits was assumed. [f] The values are from in vitro release in the vitreous as a medium. [h] The virtual delay constant for dexamethasone was determined empirically to reproduce the experimental concentration in the aqueous humor of rabbits[14]. [I] The virtual delay constant for dexamethasone in rat and human were considered as 10 and 0.5 times of the values in rabbit, respectively. The diffusion time from center of the vitreous to the borders in rats and humans is about 10 and 0.5 times of values in rabbits, respectively [13]. [k] Virtual delay constants were determined to reproduce the t_{max} values from our experimental data of pullulan-DEX in rabbits. With the same rational as the delay constant for dexamethasone, the values for rats and human were assumed to be 10 and 0.5 times of values in rabbits, respectively.

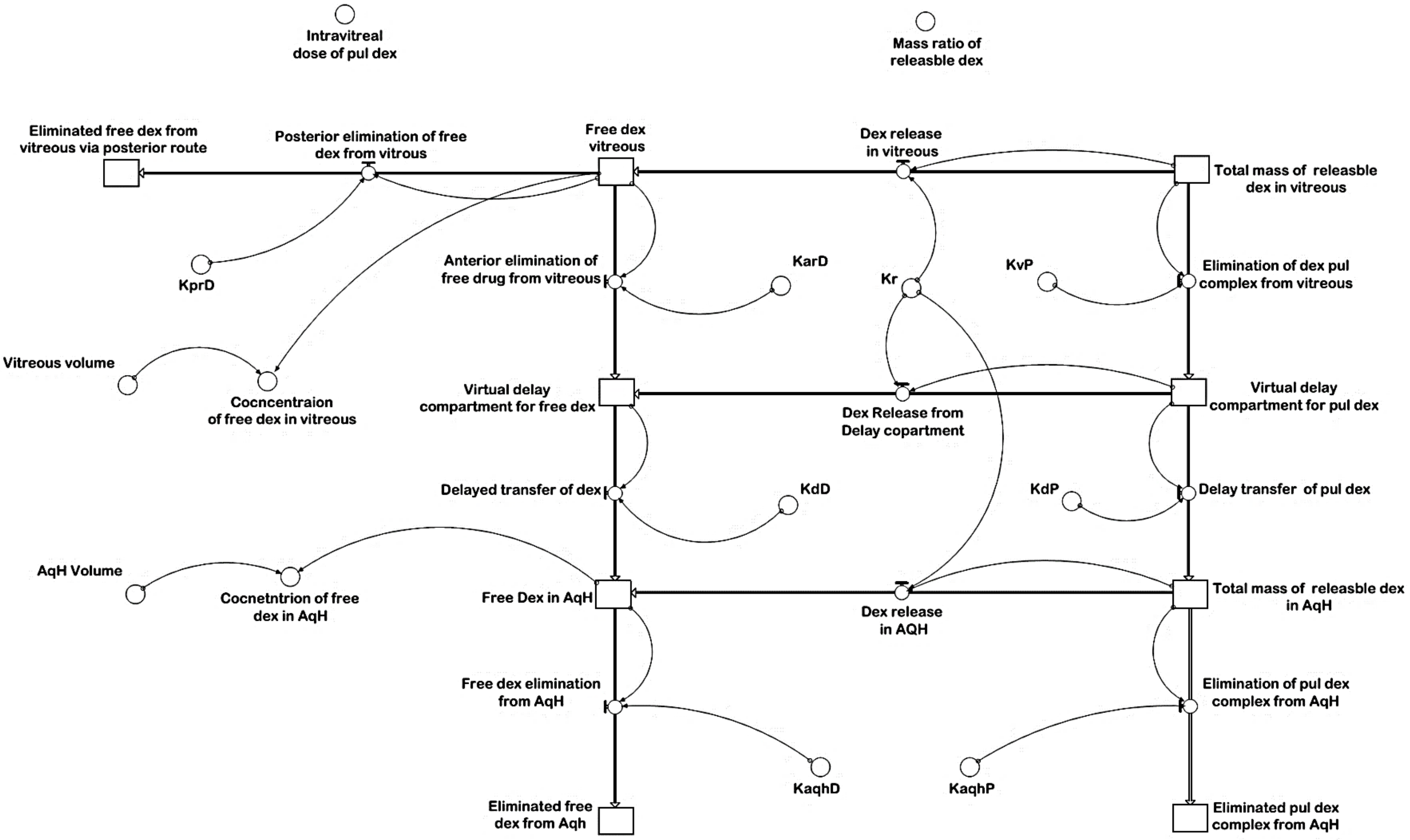

Figure S5. Stella model of the Kinetic simulations of pullulan-DEX conjugate and released dexamethasone.

SI-5 Safety on *in vivo* mice model

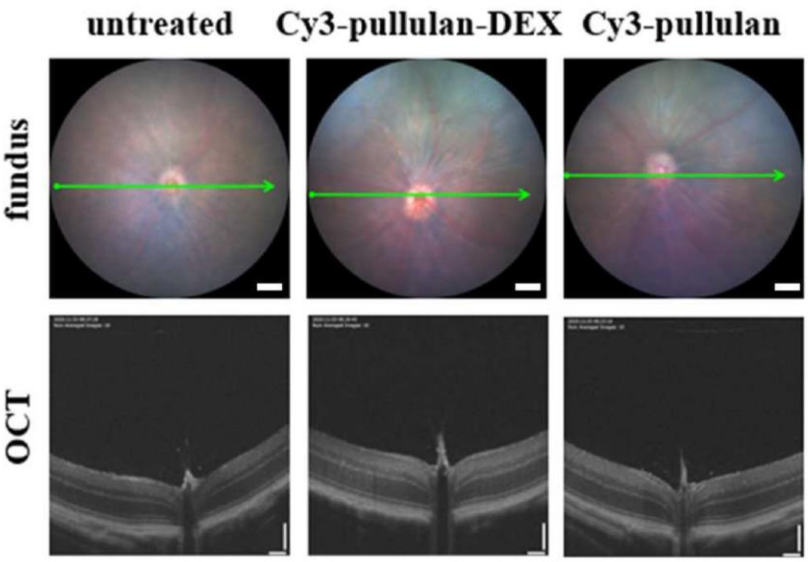

Figure S6. Fundus and optical coherence tomography (OCT) images of mouse vitreous and retina taken 24 hours after IVT injection of (1 µL of the 5 mg/mL) fluorescently labelled Cy3-pullulan-DEX and Cy3-pullulan. The untreated eye was used as control. The length of scale bar for fundus images are 200 µm. In the case of OCT, vertical and horizontal scale bars in OCT are 110 and 130 µm, respectively.

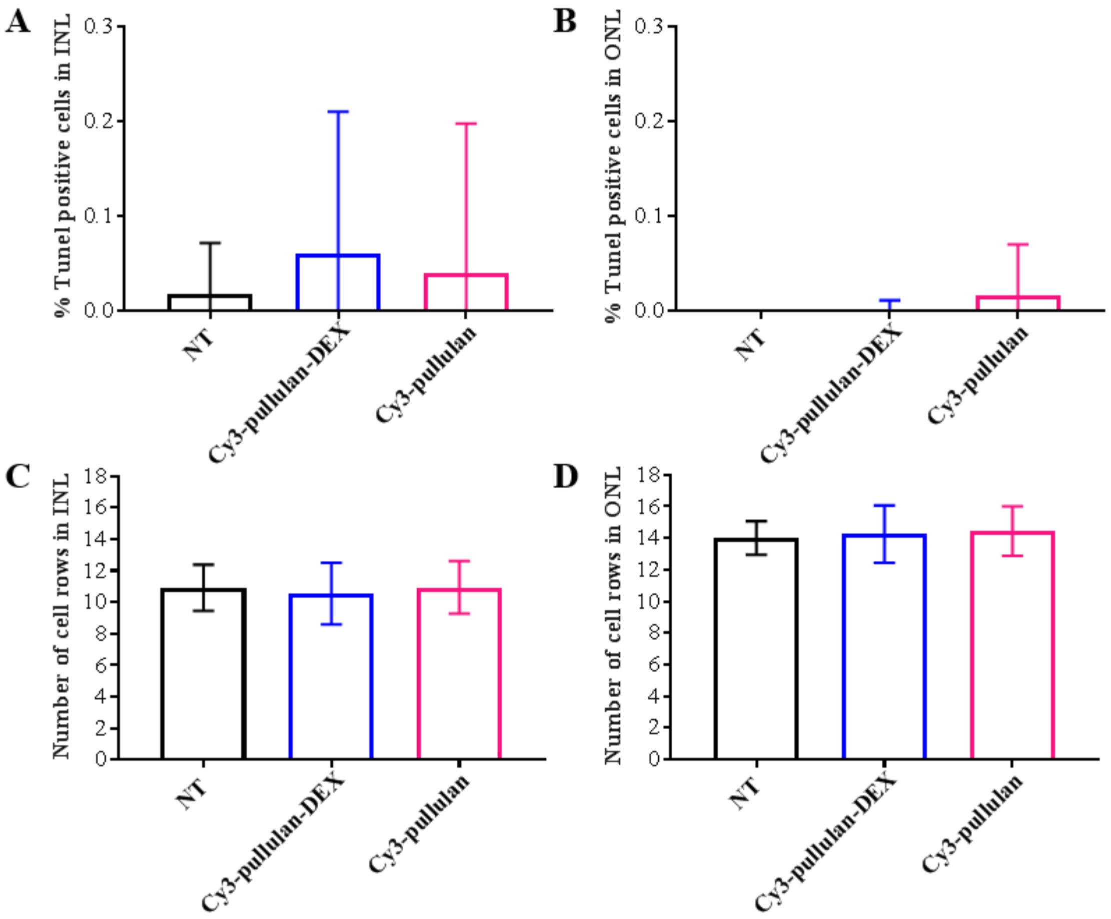

Figure S7. Two-month-old mice retinal explant analysis after IVT administration (1 µL) of the 5 mg/mL Cy3-pullulan-DEX (■) and Cy3-pullulan (■). Untreated mouse retina (■ NT) was used as control. TUNEL-positive nuclei in **A**) the inner nuclear cell layer (INL), and **B**) the outer nuclear cell layer (ONL); counted and plotted as percentage respect to all nuclei in the corresponding analyzed INL and ONL area. TUNEL-positive nuclei in the INL and ONL were counted and plotted as percentage of all nuclei in the respective INL and ONL areas. The number of cell rows in **C**) INL and **D**) ONL. Bars indicate standard deviations of means.

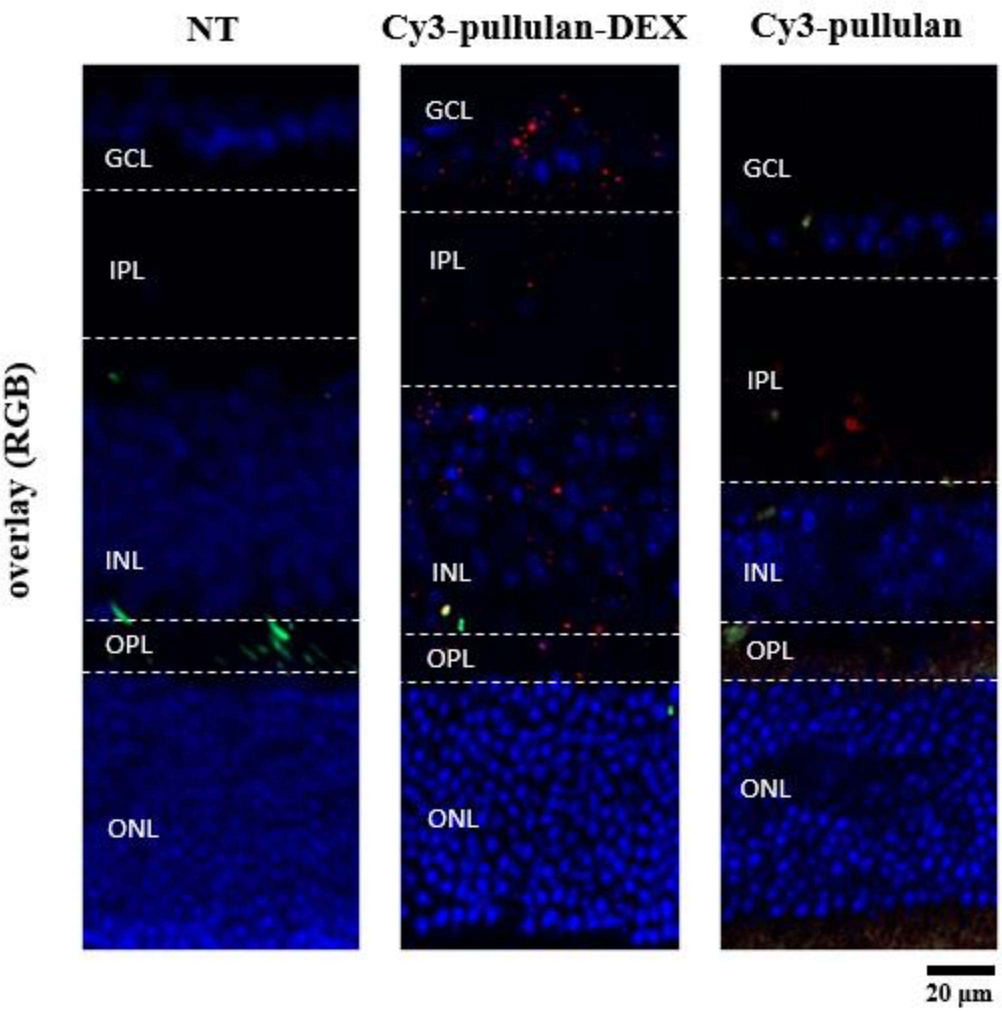

Figure S8. Images of two-month-old mice retinal sections after intravitreal administration (1 µL) of the 5 mg/mL Cy3-pullulan-DEX and Cy3-pullulan. Untreated eye (NT) was used as control. TUNEL assay (fluorescein in green), pullulan samples (cyanine3 in red) and cell nuclei (DAPI in blue). RGB: overlay of red, green and blue channels. Bar size: 20 µm. Note: selected red channels are in following Figure S9.

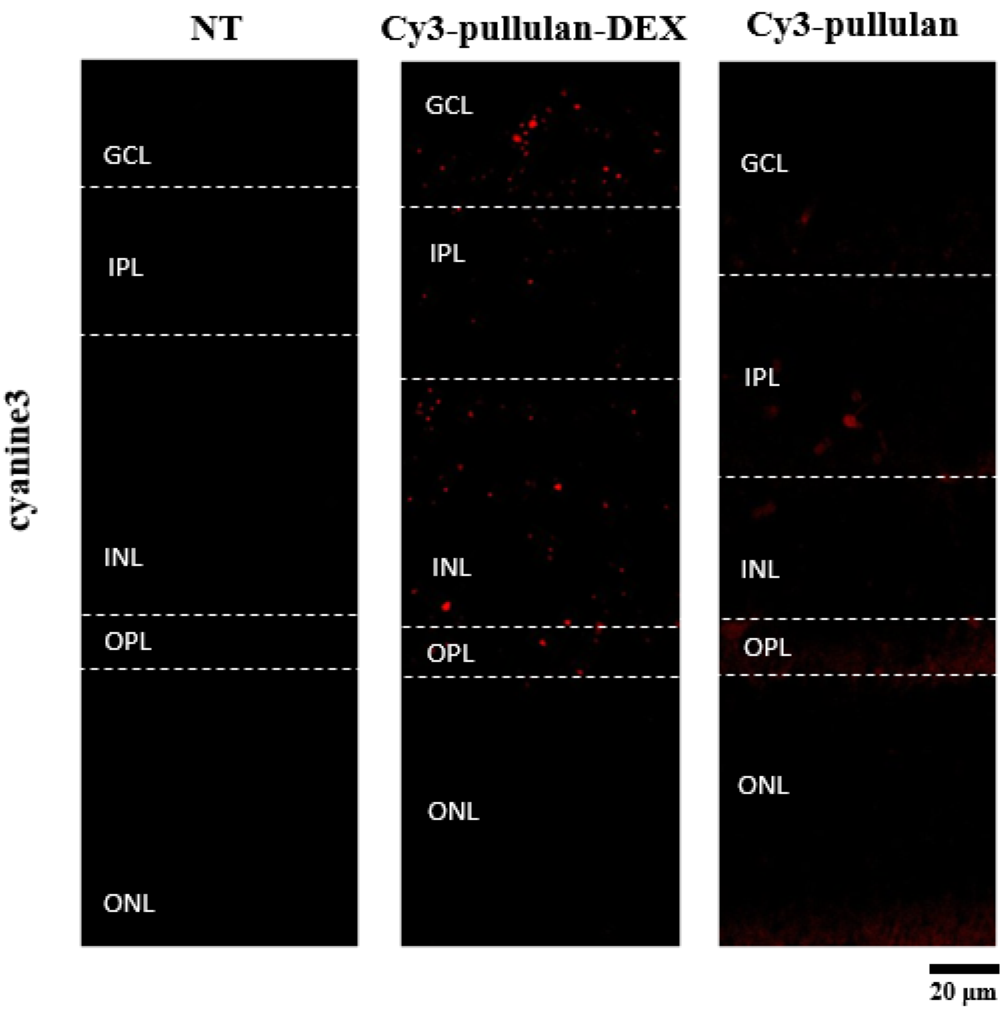

Figure S9. Images of two-month-old mice retinal sections after IVT administration (1 µL) of the 5 mg/mL Cy3-pullulan-DEX and Cy3-pullulan and untreated eye (NT) was used as control. Selected red channels referring to Figure 15 in the main text of the publication. Bar size: 20 µm.

References

1. Kicková, E.; Salmaso, S.; Mastrotto, F.; Caliceti, P.; Urtti, A. Pullulan Based Bioconjugates for Ocular Dexamethasone Delivery. *Pharm.* 2021, *13*.
2. Vineberg, J.G.; Wang, T.; Zuniga, E.S.; Ojima, I. Design, synthesis, and biological evaluation of theranostic vitamin–linker–Taxoid conjugates. *J. Med. Chem.* **2015**, *58*, 2406–2416.
3. Osakada, F.; Ooto, S.; Akagi, T.; Mandai, M.; Akaike, A.; Takahashi, M. Wnt signaling promotes regeneration in the retina of adult mammals. *J. Neurosci.* **2007**, *27*, 4210–4219.
4. Müller, B.; Wagner, F.; Lorenz, B.; Stieger, K. Organotypic cultures of adult mouse retina: Morphologic changes and gene expression. *Invest. Ophthalmol. Vis. Sci.* **2017**, *58*, 1930–1940.
5. Arango-Gonzalez, B.; Szabó, A.; Pinzon-Duarte, G.; Lukáts, Á.; Guenther, E.; Kohler, K. In vivo and in vitro development of S-and M-cones in rat retina. *Invest. Ophthalmol. Vis. Sci.* **2010**, *51*, 5320–5327.
6. Caffé, A.R.; Söderpalm, A.K.; Holmqvist, I.; van Veen, T. A combination of CNTF and BDNF rescues rd photoreceptors but changes rod differentiation in the presence of RPE in retinal explants. *Invest.*

Ophthalmol. Vis. Sci. **2001**, *42*, 275–282.
7. Gavrieli, Y.; Sherman, Y.; Ben-Sasson, S.A. Identification of programmed cell death in situ via specific labeling of nuclear DNA fragmentation. *J. Cell Biol.* **1992**, *119*, 493–501.
8. Del Amo, E.M.; Vellonen, K.S.; Kidron, H.; Urtti, A. Intravitreal clearance and volume of distribution of compounds in rabbits: In silico prediction and pharmacokinetic simulations for drug development. *Eur. J. Pharm. Biopharm.* **2015**, *95*, 215–226, doi:10.1016/j.ejpb.2015.01.003.
9. Krohne, T.I.M.U.; Eter, N.; Holz, F.G.; Meyer, C.H. Intraocular pharmacokinetics of bevacizumab after a single intravitreal injection in humans. *Am. J. Ophthalmol.* **2008**, *146*, 508–512, doi:10.1016/j.ajo.2008.05.036.
10. Zhu, Q.; Ziemssen, F.; Henke-Fahle, S.; Tatar, O.; Szurman, P.; Aisenbrey, S.; Schneiderhan-Marra, N.; Xu, X.; Grisanti, S.; Group, T.B.S. Vitreous levels of bevacizumab and vascular endothelial growth factor-A in patients with choroidal neovascularization. *Ophthalmology* **2008**, *115*, 1750–1755.
11. Mermoud, A.; Baerveldt, G.; Minckler, D.S.; Prata, J.A. Aqueous humor dynamics in rats. **1996**, 198–203.
12. Conrad, J.M.; Robinsonx, J.R. Aqueous Chamber Drug Distribution Volume Measurement in Rabbits. **1977**, *66*.
13. Civan, M.M. Transport components of net secretion of the aqueous humor and their integrated regulation. In *Current topics in membranes*; Elsevier, 1997; Vol. 45, pp. 1–24 ISBN 1063-5823.
14. Kwak, H.W.; D'Amico, D.J. Evaluation of the Retinal Toxicity and Pharmacokinetics of Dexamethasone After Intravitreal Injection. *Arch. Ophthalmol.* **1992**, *110*, 259, doi:10.1001/archopht.1992.01080140115038.
15. Gan, I.M.; Ugahary, L.C.; van Dissel, J.T.; van Meurs, J.C. Effect of intravitreal dexamethasone on vitreous vancomycin concentrations in patients with suspected postoperative bacterial endophthalmitis. *Graefe's Arch. Clin. Exp. Ophthalmol.* **2005**, *243*, 1186–1189.
16. Maurice, D.M.; Mishima, S.; Misidma, S. Ocular pharmacokinetics. In *Pharmacology of the Eye*; Springer, 1984; pp. 19–116.
17. Rosenblum, C.; Dengler, R.E.; Geoffroy, R.F. Ocular absorption of dexamethasone phosphate disodium by the rabbit. *Arch. Ophthalmol.* **1967**, *77*, 234–237.
18. Missel, P.J.; Horner, M.; Muralikrishnan, R. Simulating dissolution of intravitreal triamcinolone acetonide suspensions in an anatomically accurate rabbit eye model. *Pharm. Res.* **2010**, *27*, 1530–1546.
19. Brubaker, R.F. Flow of aqueous humor in humans [The Friedenwald Lecture]. *Invest. Ophthalmol. Vis. Sci.* **1991**, *32*, 3145–3166.
20. Hutton-Smith, L.A.; Gaffney, E.A.; Byrne, H.M.; Maini, P.K.; Schwab, D.; Mazer, N.A.; Ga, E.A.; Byrne, H.M.; Maini, P.K.; Schwab, D.; et al. A mechanistic model of the intravitreal pharmacokinetics of large molecules and the pharmacodynamic suppression of ocular vascular endothelial growth factor levels by ranibizumab in patients with neovascular age-related macular degeneration. *Mol. Pharm.* **2016**, *13*, 2941–2950, doi:10.1021/acs.molpharmaceut.5b00849.
21. Friedrich, S.; Cheng, Y.-L.; Saville, B. Drug distribution in the vitreous humor of the human eye: The effects of intravitreal injection position and volume. *Curr. Eye Res.* **1997**, *16*, 663–669.
22. Sadeghi, A.; Puranen, J.; Ruponen, M.; Valtari, A.; Subrizi, A.; Ranta, V.-P.; Toropainen, E.; Urtti, A. Pharmacokinetics of intravitreal macromolecules: Scaling between rats and rabbits. *Eur. J. Pharm. Sci.* **2021**, 105720.
23. Kidron, H.; Amo, E.M.; Vellonen, K.; Urtti, A. Prediction of the Vitreal Half-Life of Small Molecular Drug-Like Compounds. **2012**, 3302–3311, doi:10.1007/s11095-012-0822-5.
